# A new look at TFPI inhibition of factor X activation

**DOI:** 10.1371/journal.pcbi.1012509

**Published:** 2024-11-15

**Authors:** Fabian Santiago, Amandeep Kaur, Shannon Bride, Dougald Monroe, Karin Leiderman, Suzanne Sindi

**Affiliations:** 1 Department of Applied Mathematics, University of California Merced, Merced, California, United States of America; 2 Department of Applied Mathematics & Statistics, Colorado School of Mines, Golden, Colorado, United States of America; 3 UNC Blood Research Center, University of North Carolina, Chapel Hill, Chapel Hill, North Carolina, United States of America; 4 Mathematics Department, University of North Carolina, Chapel Hill, North Carolina, United States of America; 5 Computational Medicine Program, University of North Carolina, Chapel Hill, Chapel Hill, North Carolina, United States of America; University of Tennessee Health Science Center College of Medicine Memphis, UNITED STATES OF AMERICA

## Abstract

Blood coagulation is a vital physiological process involving a complex network of biochemical reactions, which converge to form a blood clot that repairs vascular injury. This process unfolds in three phases: initiation, amplification, and propagation, ultimately leading to thrombin formation. Coagulation begins when tissue factor (TF) is exposed on an injured vessel’s wall. The first step is when activated factor VII (VIIa) in the plasma binds to TF, forming complex TF:VIIa, which activates factor X. Activated factor X (Xa) is necessary for coagulation, so the regulation of its activation is crucial. Tissue Factor Pathway Inhibitor (TFPI) is a critical regulator of the initiation phase as it inhibits the activation of factor X. While previous studies have proposed two pathways—direct and indirect binding—for TFPI’s inhibitory role, the specific biochemical reactions and their rates remain ambiguous. Many existing mathematical models only assume an indirect pathway, which may be less effective under physiological flow conditions. In this study, we revisit datasets from two experiments focused on activated factor X formation in the presence of TFPI. We employ an adaptive Metropolis method for parameter estimation to reinvestigate a previously proposed biochemical scheme and corresponding rates for both inhibition pathways. Our findings show that both pathways are essential to replicate the static experimental results. Previous studies have suggested that flow itself makes a significant contribution to the inhibition of factor X activation. We added flow to this model with our estimated parameters to determine the contribution of the two inhibition pathways under these conditions. We found that direct binding of TFPI is necessary for inhibition under flow. The indirect pathway has a weaker inhibitory effect due to removal of solution phase inhibitory complexes by flow.

## Author summary

After vascular injury, a large complex network of biochemical reactions leads to clot formation to repair vessel damage. Three vital biochemical components in the early phase of this process are coagulation factor X (X), the activated coagulation factor VII and tissue factor (TF) complex (TF:VIIa) that activates X, and tissue factor pathway inhibitor (TFPI), which regulates X activation. The mechanism of this TFPI regulation remains ambiguous. Here, we develop a mathematical model of the biochemical reactions involved in this process and calibrate our model to published data from two experimental studies of factor X activation. To explain the experimental data, we find that TFPI acts through two distinct biochemical pathways: direct and indirect binding, the latter being a multi-step process involving Xa re-binding to TF:VIIa. We extend our model to study TFPI’s regulatory effects under flow and, in contrast to a previous study, demonstrate that the direct pathway of regulation is essential in the presence of flow. Incorporating our findings into larger models that include later phases of the clotting process could enhance our understanding of clotting disorders and aid in the development of targeted therapies.

## Introduction

Blood coagulation is a complex system of biochemical reactions that are required to form a clot in response to injury. The process is initiated by the exposure of tissue factor (TF) and collagen at the surface of subendothelial cells. TF exposure triggers the activation of plasma coagulation factors at the site of injury and collagen exposure recruits and activates platelets, which form an initial plug of the injury. The coagulation reactions lead to generation of the enzyme thrombin. Thrombin cleaves fibrinogen into fibrin monomers, which polymerize to form a meshwork that stabilizes the plug. The formation of a strong thrombin response within an appropriate time after injury is critical to clotting. Both over- and under-clotting are associated with life-threatening conditions [1, 2]. Therefore, it is crucial to understand the underlying dynamics of the clotting process and its regulation to prevent disease complications caused by bleeding disorders and clotting irregularities.

Mathematical modeling has been used to gain insight into the complex process of coagulation. Indeed, there are multiple models of the full coagulation pathway, as recently detailed in various reviews [3–6]. The biochemical reaction schemes and rates differ—often significantly—between models. In the most recent review, it was concluded that no existing model of thrombin generation (without flow) produced thrombin generation curves consistent with *in vitro* assays of a characterized cohort. This demonstrates there is still a substantial work to be done in identifying, establishing, and validating fully accurate and predictive mathematical models of coagulation.

Tissue factor pathway inhibitor (TFPI) plays an important regulatory role in cogaulation and is necessary for life. For example, the lack of TFPI also leads to embryonic death in mice [7, 8]. To be primed for a rapid coagulation response, clotting factors circulate in the blood in an inactive form. When the coagulation cascade begins, these factors become activated and subsequently activate other factors in the pathway. The first step in this process is the activation of coagulation factor X to its active form, factor Xa, by the complex of TF and factor VIIa (TF:VIIa). Factor Xa plays a significant role in the coagulation cascade, as it combines with factor Va to form the prothrombinase complex, where it activates prothrombin to thrombin. TFPI inhibits X activation by TF:VIIa, which affects the strength and timing of thrombin generation, and ultimately the coagulation response. Interestingly, there is a discrepancy in mathematical and experimental studies over the regulatory mechanisms of TFPI. Different mechanisms have been proposed about how this regulation is achieved [9–11].

**Fig 1:**
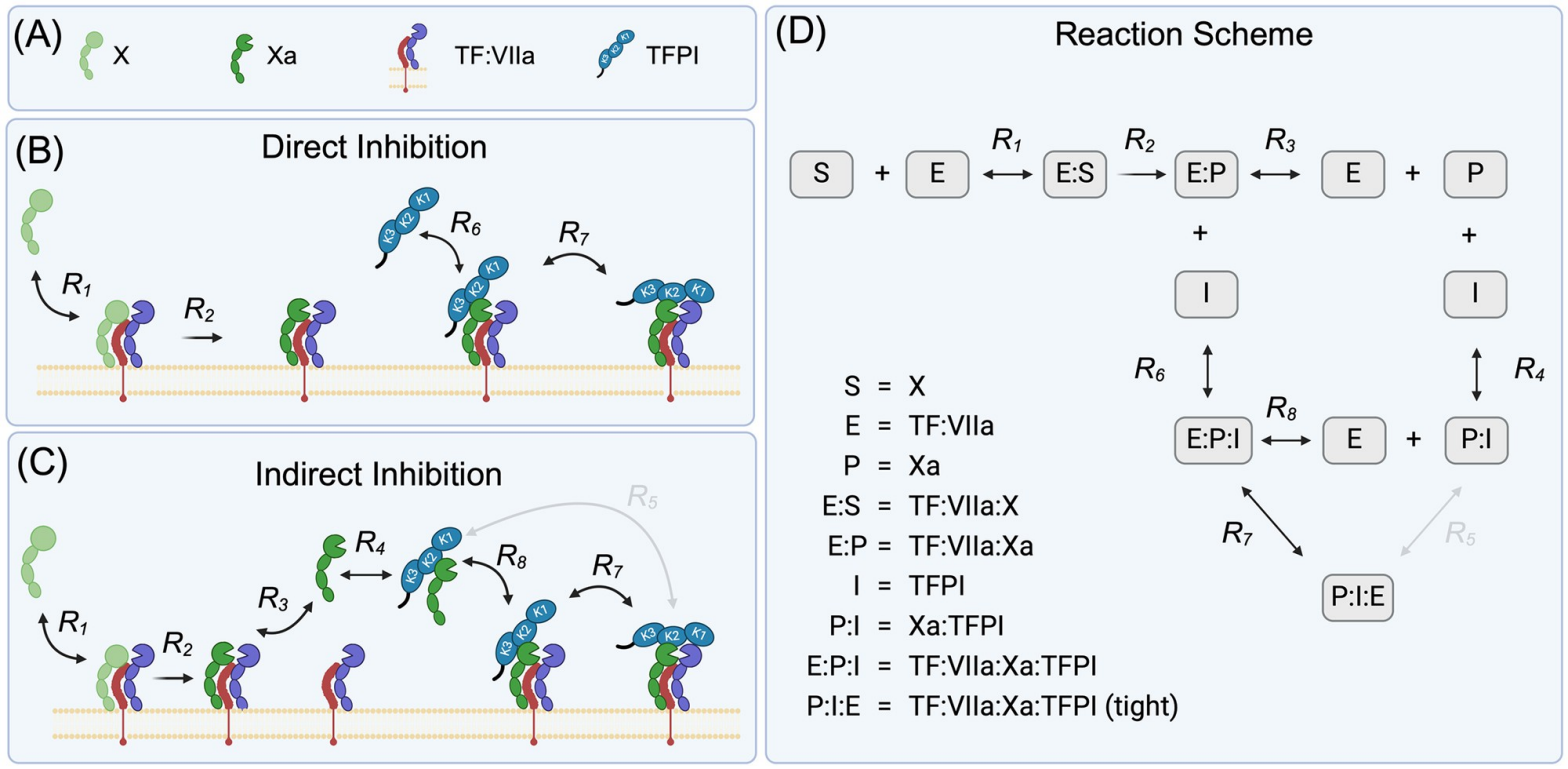
Proposed mechanisms of TFPI inhibition of factor X activation. In 1998, Baugh et al. [9] proposed a scheme with two reaction pathways for TFPI inhibition of factor X activation: direct and indirect. In our work, we consider a modification of Baugh’s scheme by restricting it to elementary reactions only and removing the multi-step reaction 5. (A) Representative structures for coagulation factors and associated labels. (B) Biochemical reactions involved in the direct inhibition of factor X activation. (C) Biochemical reactions involved in the indirect inhibition of factor X activation. (D) Schematic for the reactions involved in the activation of factor X by TF:VIIa and both the direct and indirect pathways of inhibition by TFPI.

In this work, we take a bottom-up approach to uncover the true mechanism of TFPI’s inhibtion of X activation, under both static and flow conditions. Specifically, we revisit a previously published set of experimental studies by Baugh et al. [9]. The authors proposed a scheme of TFPI inhibition of factor X activation that involved two distinct pathways (see Fig 1). Although a follow-up study suggested this scheme was inconsistent with Baugh’s data [10], we find strong support for it when using an adaptive Metropolis to fit parameters using available prior knowledge. We then add flow to our scheme and demonstrate that strong inhibition in this scenario is possible only when the direct mechanism is included. Moreover, product inhibition, which was hypothesized by a prior study [11] to be a more significant inhibitor than TFPI under flow, was shown to be negligible. In summary, using a combination of modern parameter estimation, mechanistic modeling, and experimental data, we have provided an accurate mathematical description of TFPI’s inhibory role in the initiation of coagulation.

### Biological and mathematical background of TFPI inhibition of factor X activation

TFPI consists of three distinct Kunitz-type protease inhibitor domains. (These are labeled K1, K2 and K3 in Fig 1.) It binds to Xa with the Kunitz 2 domain and to VIIa with the Kunitz 1 domain. During the activation of X by TF:VIIa, there are two intermediate and transient complexes: TF:VIIa:X and TF:VIIa:Xa, where X and Xa binding is reversible from each complex. One of TFPI’s key inhibitory functions is its reversible binding to factor Xa, which leaves open a few possiblities for how it inhibits TF:VIIa activation of X. Two distinct mechanisms were hypothesized by Baugh [9] (shown as kinetic schemes in Fig 1):

- *Direct Binding* (Fig 1A and 1B) operates through TFPI binding directly to Xa that is already bound to TF:VIIa, thereby inhibiting the enzyme activity and ultimately preventing the release of Xa.
- *Indirect Binding* (Fig 1A and 1C) involves the formation of a Xa:TFPI complex in the fluid, which can bind to TF:VIIa, blocking its ability to activate additional X.

Baugh’s hypotheses were based on a set of elegant experiments that suggested both mechanisms were at play. They did not determine kinetic rates constants for direct binding but did measure apparent rates for inhibition through indirect binding. A subsequent theoretical study considered four possible kinetic schemes, including the Baugh scheme, and compared their optimized model outputs with the Baugh experiments [10]. The authors concluded that it was impossible to fit the experimental data with Baugh’s proposed scheme, but that their own hypothesized scheme did match the data. In our opinion, while the alternative scheme may have represented the data, it does not represent the current biochemical knowledge of the reactions considered.

Indeed, mathematical models of coagulation include different reaction schemes (and reaction rates) for TFPI. We note that the direct binding mechanism for TFPI is not included in many mathematical models of the full coagulation system, including, the lipid-dependent model from Bungay and colleagues [12] and even our own models of coagulation under flow [13–15]. In fact, many models examined in the review [6] consider the formation of a stable quaternary complex involving TF, TFPI, factor VIIa, and factor Xa, but generally do not consider an intermediate configuration prior to reaching this stable quaternary complex. The kinetic rates for all models were shown in the supplemental S1 Table. The rates vary significantly across different studies, including [16–21]. The variations in the rate constants revealed differences in predictions made by the models in the review.

In [18, 22], Danforth and colleagues performed comprehensive sensitivity analyses on a static model of coagulation (i.e., a well-mixed biochemical system with no flow in or out). They determined which coagulation factors most strongly influenced the final level of thrombin and observed that TFPI, along with another inhibitor antithrombin, was the most important contributor to the final level of thrombin. Indeed, the importance of TFPI in static coagulation is also affirmed through observations from *in vitro* experiments such as thrombin generation assays (TGA) [23].

Of course, coagulation *in vivo* occurs under flow with continual supply and removal of factors from the injury site, where TF:VIIa remains fixed because it is bound to the vessel wall at the site of the injury. A mathematical study by Fogelson and Tania [11] considered a model of coagulation under flow (i.e., where a well-mixed reaction zone has factors continually flowing in and out) where the indirect binding and some form of the direct binding mechanisms were considered. The direct binding mechanism was investigated by systematically decreasing the dissocation constant between TF:VIIa and Xa to test whether product inhibition or TFPI binding was a more prominent form of inhibition. Interestingly, they concluded that flow played a far more critical role in thrombin regulation than biochemical inhibitors such as TFPI (regardless of binding scheme), antithrombin (AT), and activated protein C. In our opinion, the scheme and kinetic rates used within that study did not represent the true ones that we will soon discuss in this study. A later study with a similar kinetic model of coagulation under flow [24] conducted a comprehensive sensitivity analysis and reaffirmed the that thrombin was not sensitive to biochemical inhibitors such as AT and TFPI. However, in that case, the model only considered the indirect binding mechanism. Another theoretical study of coagulation did find TFPI to inhibit thrombin generation overall, but the mechanism was based on the aforementioned alternative TFPI scheme [10]. Collectively, these findings suggest that TFPI’s role in coagulation is not adequately represented in our current mathematical models.

## Results

### Modeling TFPI inhibition of factor X activation by TF:VIIa

Our kinetic scheme is based heavily on “Scheme II” presented by Baugh [9] for TFPI inhibition of factor X activation. Their full scheme includes eight reactions, and for ease of comparison, we use their notation numbering. We have chosen to remove reaction 5 from the Baugh scheme as it involves two simultaneous binding events: Xa with TF:VIIa and TFPI with TF:VIIa (through the Kunitz 1 domain). (The likelihood of the Xa:TFPI complex undergoing two (effectively) simultaneous binding events with the TF:VIIa complex to directly from the quaternary complex TF:VIIa:Xa:TFPI (tight) (Reaction 5) is extremely small compared to that of the likelihood of forming an TF:VIIa:Xa:TFPI complex through a single binding event (Reaction 8)). While some mathematical models do not distinguish between the quaternary complex with and without Kunitz 1 bound to TF:VIIa, we believe this distinction is important. There is ample evidence that TFPI’s Kunitz 1 domain is crucial for coagulation.

In [25], researchers observed through a series of point mutation studies that the Kunitz 1 domain binds to VIIa, and TFPI lacking a functional Kunitz 1 domain cannot block TF:VIIa activity. As such, the role and binding of Kunitz 1 to VIIa appear critical for regulating TF:VIIa activity. This has also been observed in other studies of truncated TFPI lacking the Kunitz 1 domain [26, 27]. Together, this suggests to us that the final binding event of the Kunitz 1 domain of TFPI is critical for the inhibition of TF:VIIa.

We use our modified kinetic scheme (Fig 1) to define a system of ordinary differential equations (ODEs) (Eq (1)). These ODEs track the time-varying concentrations of all nine biochemical species (*E* = TF:VIIa, *S* = X, E:S = TF:VIIa:X, E:P = TF:VIIa:Xa, *P* = Xa, *I* = TFPI, P:I = Xa:TFPI, E:P:I = TF:VIIa:Xa:TFPI, P:I:E = TF:VIIa:Xa:TFPI (tight)) as they evolve in a well-mixed solution:

d[E]dt=-k+1[E][S]+k-1[E:S]-k+3[E][P]+k-3[E:P]-k+5[E][P:I]+k-5[P:I:E]-k+8[E][P:I]+k-8[E:P:I] (1a)

$$\frac{d[S]}{dt}=-k_{+1}\left[E\right]\left[S\right]+k_{-1}\left[\text{E:S}\right]$$ (1b)

$$\frac{d[\text{E:S}]}{dt}=k_{+1}\left[E\right]\left[S\right]-k_{-1}\left[\text{E:S}\right]-k_{+2}\left[\text{E:S}\right]$$ (1c)

$$\frac{d[\text{E:P}]}{dt}=k_{+2}\left[\text{E:S}\right]+k_{+3}\left[E\right]\left[P\right]-k_{-3}\left[\text{E:P}\right]-k_{+6}\left[\text{E:P}\right]\left[I\right]+k_{-6}\left[\text{E:P:I}\right]$$ (1d)

$$\frac{d[P]}{dt}=-k_{+3}\left[E\right]\left[P\right]+k_{-3}\left[\text{E:P}\right]-k_{+4}\left[P\right]\left[I\right]+k_{-4}\left[\text{P:I}\right]$$ (1e)

$$\frac{d[I]}{dt}=-k_{+4}\left[P\right]\left[I\right]+k_{-4}\left[\text{P:I}\right]-k_{+6}\left[\text{E:P}\right]\left[I\right]+k_{-6}\left[\text{E:P:I}\right]$$ (1f)

d[P:I]dt=k+4[P][I]-k-4[P:I]-k+5[E][P:I]+k-5[P:I:E]-k+8[E][P:I]+k-8[E:P:I] (1g)

$$\begin{array}{cc} \frac{d[\text{E:P:I}]}{dt} & =k_{+6}\left[\text{E:P}\right]\left[I\right]-k_{-6}\left[\text{E:P:I}\right]-k_{+7}\left[\text{E:P:I}\right]+k_{-7}\left[\text{P:I:E}\right]\\ & \;+k_{+8}\left[E\right]\left[\text{P:I}\right]-k_{-8}\left[\text{E:P:I}\right] \end{array}$$ (1h)

d[P:I:E]dt=k+5[E][P:I]-k-5[P:I:E]+k+7[E:P:I]-k-7[P:I:E]. (1i)

All nine species (Eqs (1a) to (1i)) are tracked in units of nanomolar (nM). The forward reaction rate constants, denoted as *k*_(+)_, have units of (nM)^−1^s^−1^, while the reverse reaction rate constants, denoted as *k*_(−)_, have units of s^−1^. However, *k*_+2_ and *k*_+7_ are exceptions and have units of s^−1^. This is because *k*_+2_ represents the catalytic rate, and *k*_+7_ represents the probability per unit time that the E:P:I complex will undergo a conformational change to the stable P:I:E complex.

### Fitting kinetic parameters to experimental data

**Fig 2:**
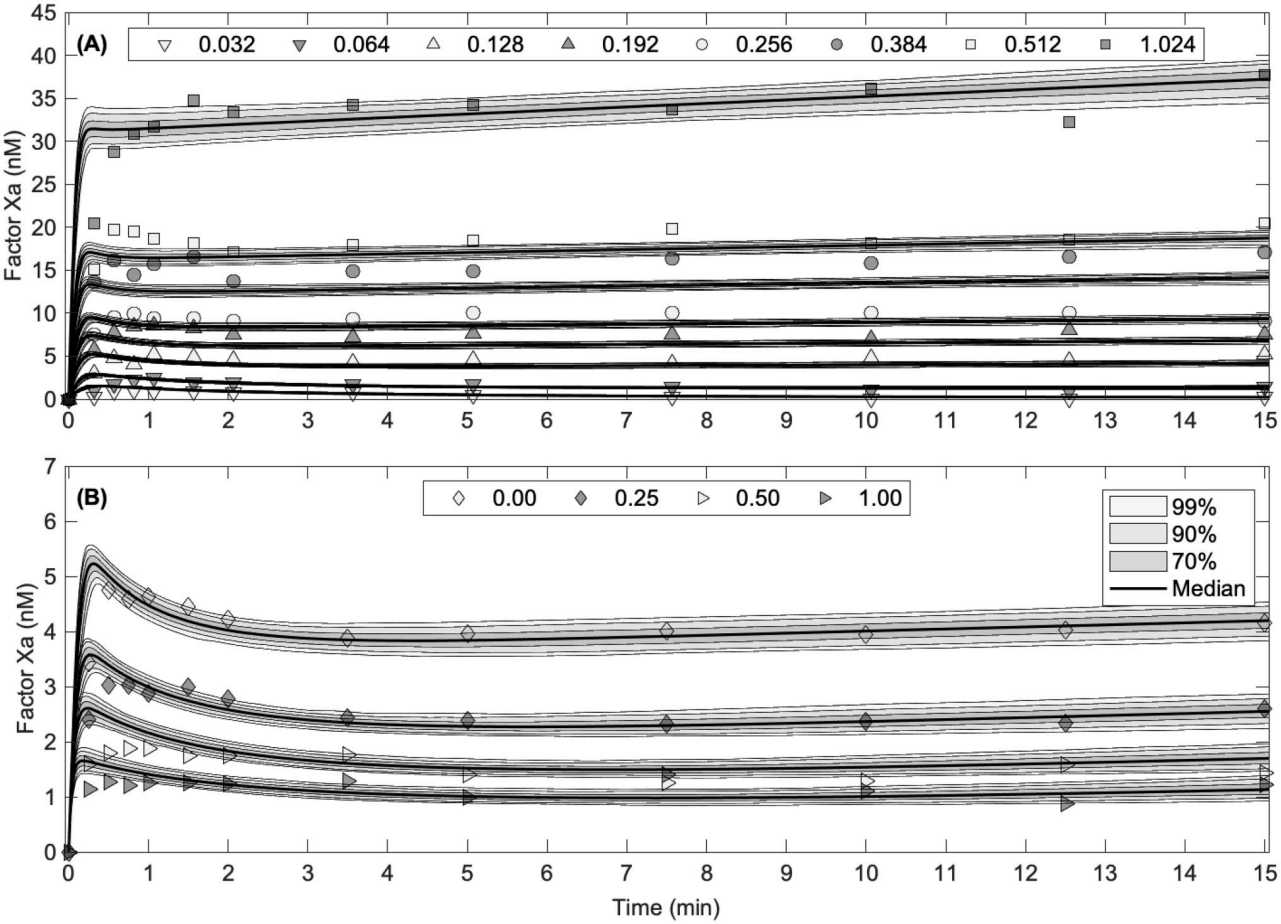
Experimental measurements and uncertainty in model predictions of factor X activation. (A) Factor X (170 nM) activated by TF:VIIa (0.032 to 1.024 nM) in the presence of TFPI (2.4 nM). (B) Factor X (170 nM) activated by TF:VIIa (0.128 nM) in the presence of TFPI (2.4 nM), preincubated with factor Xa (0.00 to 1.00 nM). Data extracted from Figure 3B of [9]. The curves show model predictions using median and literature values presented in Table 1, and the uncertainty in model predictions using posterior estimates (see S1 and S2 Figs): 70%, 90%, and 99% credible intervals about the median model prediction. Note that there is a replicate experimental condition between both experiments. The Xa = 0 nM pre-incubation condition in Experiment Two (B) matches the TFPI 0.128 nM curve in Experiment One (A).

We extracted experimental data from Figs 2A and 3B of [9] using the online tool [28]. The extracted data points are given in S1 Text (see Methods section). The focus was on the formation of factor Xa in the presence of tissue factor pathway inhibitor (TFPI) under two distinct experimental conditions. We refer to these as Experiment One Fig 2(A) and Experiment Two Fig 2(B). Below, we describe the setup for each experiment:

- **Experiment One**: Measures factor Xa generation under varying concentrations of the enzyme TF:VIIa (ranging from 0.032 to 1.024 nM) with fixed amounts of factor X (170 nM) and TFPI (2.5 nM), as shown in Fig 2(A).
- **Experiment Two**: Consists of a *pre-incubation* phase and a *post-incubation* phase to explore the effects of pre-formed complexes on factor X activation. In the *pre-incubation phase*, TFPI is fixed at 2.4 nM, and the concentration of Xa is varied (from 0 to 1 nM). The mixture is incubated for 2 hours. In the *post-incubation phase*, the resulting solution is combined with factor X (170 nM) and TFPI (0.128 nM), as shown in Fig 2(B).

In fitting models to the data, we employ a Bayesian approach and use prior knowledge of known kinetic parameters from the literature (see Table 1). More specifically, we use the dissociation constant for Xa and TF:VIIa (*k*_+3_/*k*_−3_ = *K*_3_ = 520 nM) reported by Lu [29] and the Michaelis constant ((*k*_+1_ + *k*_2_)/*k*_−1_ = *K*_*M*_ = 238 nM) for X activation by TF:VIIa reported by Baugh [9]. The *K*_*D*_ represents the affinity between an enzyme and its substrate, a lower *K*_*D*_ indicates a higher affinity, meaning the enzyme and substrate bind more tightly. The Michaelis constant reflects the substrate concentration the reaction rate is at half of its maximum value.

**Table 1:** Estimated rate and dissociation constants. This table lists each reaction number alongside its corresponding biochemical equation, kinetic factors, and parameter units. Median values and literature sources are provided, with the last column showing 95% credible intervals (CI) for the estimated kinetic factors. This comprehensive data allows for a detailed analysis of reaction dynamics under flow. Note that reaction number five was nullified in this work. Additionally, a star (*) in the 95% CI column indicates that the corresponding factor was fixed to the specified literature value. ^†^The dissociation constant *K*_*D*,4_ was recomputed separately from data presented in [9]; see supplemental S1 Text.

Reaction Number	Biochemical Equation	Kinetic Factor	Units	Median/Literature	95% CI
1	$E+S\overset{k_{+1}}{\underset{k_{-1}}{⇌}}E:S$	*k*_+1_	(nM)^−1^s^−1^	0.51	(8.94 × 10^−2^, 0.96)
		*k*_−1_	s^−1^	104.30	(5.10, 212.37)
		*K*_*M*_	nM	238 [9]	*
		*K*_*D*,1_	nM	205.85	(56.82, 222.11)
2	$E:S\overset{k_{+2}}{\to }E:P$	*k*_+2_	s^−1^	16.23	(12.61, 19.77)
3	$E:P\overset{k_{-3}}{\underset{k_{+3}}{⇌}}E+P$	*k*_+3_	(nM)^−1^s^−1^	0.16	(5.17 × 10^−2^, 0.30)
		*k*_−3_	s^−1^	81.18	(26.90, 153.42)
		*K*_*D*,3_	nM	520 [29]	*
4	$P+I\overset{k_{4}}{\underset{k_{-4}}{⇌}}P:I$	*k*_+4_	(nM)^−1^s^−1^	3.67 × 10^−3^	(2.68 × 10^−3^, 5.07 × 10^−3^)
		*k*_−4_	s^−1^	9.64 × 10^−5^	(7.04 × 10^−5^, 1.33 × 10^−4^)
		*K*_*D*,4_	nM	2.63 × 10^−2†^	-
5	$E+P:I\overset{k_{+5}}{\underset{k_{-5}}{⇌}}P:I:E$	*k*_+5_	(nM)^−1^s^−1^	-	-
		*k*_−5_	s^−1^	-	-
		*K*_*D*,5_	nM	-	-
6	$E:P+I\overset{k_{+6}}{\underset{k_{-6}}{⇌}}E:P:I$	*k*_+6_	(nM)^−1^s^−1^	0.56	(0.19, 0.95)
		*k*_−6_	s^−1^	25.16	(0.81, 85.11)
		*K*_*D*,6_	nM	46.13	(1.43, 257.03)
7	$E:P:I\overset{k_{+7}}{\underset{k_{-7}}{⇌}}P:I:E$	*k*_+7_	s^−1^	360.92	(97.52, 491.41)
		*k*_−7_	s^−1^	7.36 × 10^−3^	(3.21 × 10^−3^, 9.86 × 10^−3^)
		*K*_*R*,7_	-	2.00 × 10^−5^	(8.28 × 10^−6^, 8.71 × 10^−5^)
8	$E+P:I\overset{k_{+8}}{\underset{k_{-8}}{⇌}}E:P:I$	*k*_+8_	(nM)^−1^s^−1^	0.95	(0.80, 1.00)
		*k*_−8_	s^−1^	16.03	(0.49, 71.82)
		*K*_*D*,8_	nM	17.15	(0.53, 76.86)

We employ an adaptive Metropolis approach to estimate unknown parameters based on prior knowledge, as described in the Methods section. In Table 1, we show the median value and provide a 95% credibility interval for all parameters, formed using the resulting posterior estimates (see S1 and S2 Figs). We assumed no error in the initial conditions and that the experimental outputs are independent measurements that vary normally around the true solution. We assume the error at each point is proportional to the model solution. We used a uniform prior over a finite range set for each parameter. This finite range is based on prior reported values of kinetic parameters (e.g., *K*_*D*_, *K*_*M*_), known relationships, or biophysical limits on diffusion (a maximum value for the forward rate *k*_(+)_). While there are no known biophysical limits on unbinding, we assumed a maximum value of 500 s^−1^ for the reverse reaction rate *k*_(−)_ and the conformational change rate *k*_7_. Note that when presenting rates we also list the *K*_*R*,7_ = *k*_+7_/*k*_−7_. This dimensionless quantity demonstrates the strength of the *P*: *I*: *E* tight complex. (See Methods for more information.)

In Fig 2, we show our model predictions for each experiment type using the median parameter values presented in Table 1, along with 70%, 90%, and 99% credible intervals over time for each prediction. This uncertainty in the predictions is formed using the model solutions computed from the resulting MCMC chain parameter estimates. We note that the resulting output curves are consistent with both experiment types. Therefore, in contrast to Pantaleev [10], we conclude that both experiments are consistent with our biochemical model and prior knowledge of kinetic parameters.

### TFPI inhibition of factor X activation by TF:VIIa under flow

With a set of kinetic rates aligned with our static model formulation (Table 1), we next turned our attention to the influence of flow on TFPI-mediated inhibition. It is important to note that previous research in a flow-based model identified product inhibition as a more potent inhibitor than TFPI in the presence of flow [11]. This finding prompts us to further investigate how flow conditions modify the inhibitory dynamics observed in static conditions.

**Fig 3:**
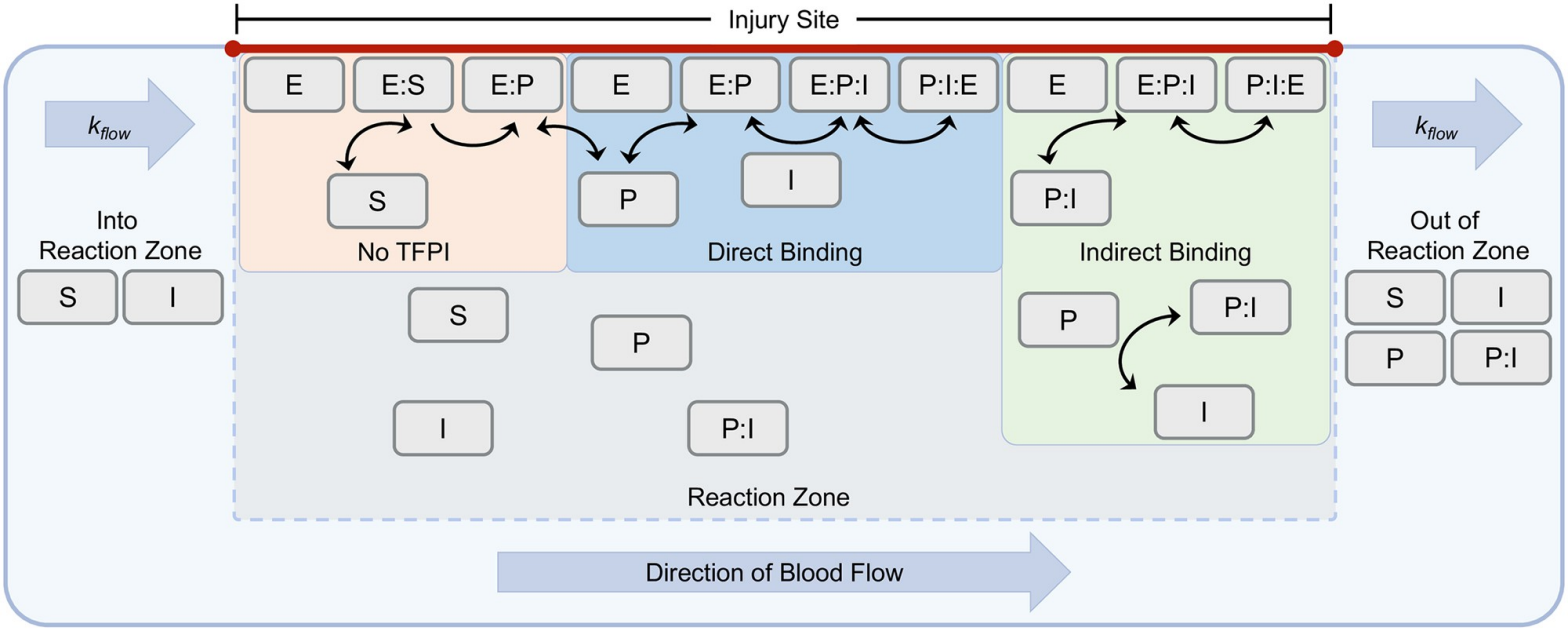
Inhibition of factor X activation under flow. During the early phase of the coagulation cascade, clotting factor X (denoted as S) is activated by the surface-bound TF:VIIa complex (denoted as E), as shown in the pathway labeled ‘No TFPI.’ Tissue Factor Pathway Inhibitor (TFPI, denoted as I), also brought in by flow, inhibits this activation. Initially, TFPI forms the transient E:P:I complex with the E complex and activated factor X (denoted as P). This complex rapidly undergoes a conformational change to form a stable P:I:E complex, depicted as ‘Direct Binding.’ Additionally, TFPI can inhibit factor X activation through a second mechanism, starting with the formation of the P:I complex. This complex subsequently binds to the E complex, forming the transient E:P:I complex that also transitions into the stable P:I:E configuration, as illustrated in the ‘Indirect Binding’ pathway.

First, we generalized our static model to include flow in and out of a reaction zone (see Fig 3 and Eq (2)). This simplified model assumes that all biochemical species are well mixed within this zone. Consequently, the clotting factor concentration dynamics result from interactions with other species through biochemical reactions in the fluid, at the injury site, and are influenced by transport into and out of the reaction zone by flow.

We then developed a metric to quantify TFPI inhibition and studied the inhibitory strength of TFPI under the presence (or absence) of different reactions in our kinetic scheme. We found that, over a broad range of flow rates, strong inhibition by TFPI is possible under our model, but only when the direct mechanism is present.

#### Flow model of TFPI inhibition of factor X activation by TF:VIIa

In Eq (2), we generalize the model given by Eq (1) to account for dynamics under flow. Under physiological flow conditions, TF:VIIa (E) is modeled as preformed and anchored within the locus of the injury site and is not affected by flow. The clotting factors factor X (S_up_) and the inhibitor TFPI (I_up_) are brought into the reaction zone by flow, while factor X (S), factor Xa (P), TFPI (I), and the Xa:TFPI (P:I) complex are transported out of the reaction zone by flow. In our analyses, we set I_up_ = 170 nM [30] and S_up_ = 2.4 nM [31].

d[E]dt=-k+1[E][S]+k-1[E:S]-k+3[E][P]+k-3[E:P]-k+5[E][P:I]+k-5[P:I:E]-k+8[E][P:I]+k-8[E:P:I] (2a)

$$\frac{d[S]}{dt}=-k_{+1}\left[E\right]\left[S\right]+k_{-1}\left[\text{E:S}\right]+k_{flow}\left(\left[S_{\text{up}}\right]-\left[S\right]\right)$$ (2b)

$$\frac{d[\text{E:S}]}{dt}=k_{+1}\left[E\right]\left[S\right]-k_{-1}\left[\text{E:S}\right]-k_{+2}\left[\text{E:S}\right]$$ (2c)

$$\frac{d[\text{E:P}]}{dt}=k_{+2}\left[\text{E:S}\right]+k_{+3}\left[E\right]\left[P\right]-k_{-3}\left[\text{E:P}\right]-k_{+6}\left[\text{E:P}\right]\left[I\right]+k_{-6}\left[\text{E:P:I}\right]$$ (2d)

$$\frac{d[P]}{dt}=-k_{+3}\left[E\right]\left[P\right]+k_{-3}\left[\text{E:P}\right]-k_{+4}\left[P\right]\left[I\right]+k_{-4}\left[\text{P:I}\right]-k_{flow}\left[P\right]$$ (2e)

$$\begin{array}{cc} \frac{d[I]}{dt} & =-k_{+4}\left[P\right]\left[I\right]+k_{-4}\left[\text{P:I}\right]-k_{+6}\left[\text{E:P}\right]\left[I\right]+k_{-6}\left[\text{E:P:I}\right]\\ & \;+k_{flow}\left(\left[I_{\text{up}}\right]-\left[I\right]\right) \end{array}$$ (2f)

d[P:I]dt=k+4[P][I]-k-4[P:I]-k+5[E][P:I]+k-5[P:I:E]-k+8[E][P:I]+k-8[E:P:I]-kflow[P:I] (2g)

$$\begin{array}{cc} \frac{d[\text{E:P:I}]}{dt} & =k_{+6}\left[\text{E:P}\right]\left[I\right]-k_{-6}\left[\text{E:P:I}\right]-k_{+7}\left[\text{E:P:I}\right]+k_{-7}\left[\text{P:I:E}\right]\\ & \;+k_{+8}\left[E\right]\left[\text{P:I}\right]-k_{-8}\left[\text{E:P:I}\right] \end{array}$$ (2h)

d[P:I:E]dt=k+5[E][P:I]-k-5[P:I:E]+k+7[E:P:I]-k-7[P:I:E] (2i)

#### Quantifying TFPI inhibition under flow

Following [11, 32], we assumed an injury length of *L* = 10 *μ*M and called this region the reaction zone. Clotting factors are transported into and out of the reaction zone by a combination of flow and diffusion, represented by a mass transfer coefficient, called the flow rate, denoted by *k*_*flow*_. The value of *k*_*flow*_ (s^−1^) is computed using Eq (3) for a particular vessel type, where *V* is the midstream velocity, *L* is the injury length, *D* is the molecular diffusivity, and *R* is the vessel radius [32]:

$$\begin{array}{c} k_{flow}=\frac{3}{4}(\frac{V^{2}D}{{\left(RL\right)}^{2}}{)}^{1/3}. \end{array}$$ (3)

We determined a range of physiologically relevant flow rates (*k*_*flow*_) for our analysis that would encompass various vessel types by applying Eq (3) to reported midstream velocities and diameters for various blood vessels, from arteries to veins [33]. Given that the molecules of interest have molecular weights in the range of 41 kDa for TFPI [31] to 56 kDa for factor X [34], we set the molecular diffusivity to *D* = 50 *μ*m^2^/s based on reported values in [35]. We found that the range from *k*_*flow*_ = 10^−3^ to 10^3^ s^−1^ captures the flow rates across these vessel types.

Our goal is to quantify the inhibition of factor X activation by TFPI and factor Xa under flow through the following pathways of inhibition present in our flow model (Eq (2)):

- **No TFPI (NI)**: This pathway involves the inhibition of the enzyme by activated factor X (denoted Xa) in the absence of TFPI. It includes reactions 1 through 3, but not reactions 4 through 8.
- **Direct Binding (DB)**: This pathway involves the direct formation of the stable complex P:I:E through reactions 1 through 3 and reactions 6 through 8, excluding reaction 4.
- **Indirect Binding (IB)**: This pathway involves the indirect formation of the stable complex P:I:E through reactions 1 through 4 and reactions 7 and 8, excluding reaction 6.
- **Direct and Indirect Binding (DIB)**: In this case, all inhibition pathways are present (see Fig 3).

To assess the impact of distinct mechanisms under varying flow conditions, we utilized median rates from Table 1 and nullified rates related to reactions absent in each inhibition pathway. Our goal is to quantify the inhibition by determining the concentration of the enzyme that remains functional in the reaction zone (see Fig 3). Specifically, we quantify the total concentration of the TF:VIIa (E) and the TF:VIIa:X (E:S) complex, as shown in Eq (4):

$$\begin{array}{c} E_{functional}=\left[E\right]+\left[\text{E:S}\right]. \end{array}$$ (4)

Tracking the concentrations of E and E:S provides insights into the coagulation cascade and the mechanisms through which TFPI and factor Xa regulate TF:VIIa activity. A measurement equal to the initial enzyme amount, *E*_*functional*_ = *E*(0), indicates no inhibition by TFPI or Xa. Conversely, *E*_*functional*_ = 0 denotes complete inhibition. Next, we analyzed the steady-state behavior of this system.

#### Strong inhibition only possible with direct binding pathway

**Fig 4:**
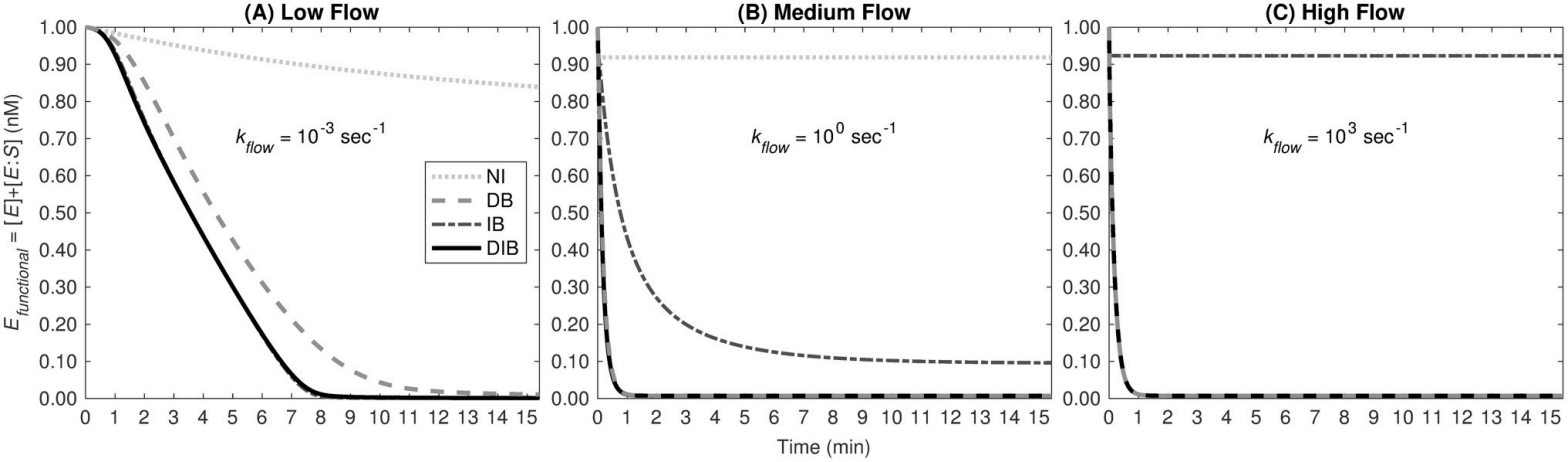
Functional enzyme over time by inhibition pathway. Concentration of functional enzyme over time by pathway of inhibition, for (A) low flow (*k*_*flow*_ = 10^−3^ s^−1^), (B) medium flow (*k*_*flow*_ = 10^0^ s^−1^), and (C) high flow (*k*_*flow*_ = 10^3^ s^−1^).

**Fig 5:**
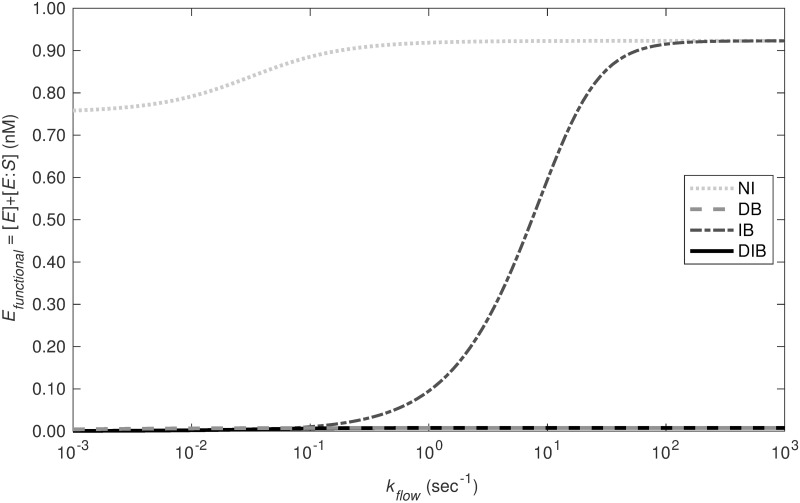
Functional enzyme at steady state over flow rate. For each model, the steady state concentration of functional enzyme ([*E*] and [*E:S*]) is presented for flow rates from 10^−3^ (Low Flow) to 10^3^ sec^−1^ (High Flow).

Complete inhibition is only observed when the direct binding pathway is active. In Fig 4, we examined the time dynamics of *E*_*functional*_ under three inhibition pathways at low (10^−3^ s^−1^), medium (10^0^ s^−1^), and high (10^3^ s^−1^) flow rates. Under nearly static conditions (low flow), as illustrated in Fig 4(A), the No TFPI (NI) pathway alone fails to fully inhibit the functional enzyme within 15 minutes. According to Fig 5, without TFPI, functional enzyme levels do not drop below approximately 0.76 nM. At low flow, the Indirect Binding (IB) pathway achieves complete inhibition more rapidly than the Direct Binding (DB) pathway. Under medium flow—more biologically pertinent conditions—the inhibition is quicker through the IB and DB pathways, though complete inhibition only occurs via the DB pathway. Here, the NI pathway is ineffective, leaving around 0.92 nM of functional enzyme at steady state. At high flow rates, the behavior is similar; complete inhibition is achievable solely through the DB pathway. Fig 5 demonstrates that with increasing flow rates, the inhibitory effects of the NI and IB pathways diminish, while the DB pathway’s ability to completely inhibit remains substantial and unaffected.

### Alternative model

We have demonstrated that the biochemical scheme derived from Scheme II in [9] effectively explains the experimental data on factor X activation (Fig 1). Building on this foundation, we now present evidence that a stable inhibitor complex is essential for the observed reactions. Furthermore, we establish that TFPI acts as a dominant inhibitor both in static conditions and under flow.

#### Necessity of stable inhibitory complex P:I:E

Having identified a model that adequately explains the experimental data (Fig 1), we now explore an alternative scheme. A noteworthy aspect of Scheme II proposed in [9] is the transition from the complex E:P:I to a more stable complex P:I:E, characterized by a significantly prolonged half-life. This transition, often omitted in mathematical models [17–21], prompted us to investigate if other plausible explanations could be formulated without this complex structure. By removing reaction 7 and re-estimating our kinetic rates using the adaptive Metropolis method, we observed that the model compensates for the absence of this reaction by enhancing the irreversibility of reactions 6 and 8 (see Table 1), effectively suggesting an emergent stable inhibitory complex (as detailed in Table 2, and S4 and S5 Figs). We note that various coagulation models incorporate the biochemistry proposed by this alternate model, including those outlined in recent studies [9, 17–19, 21].

**Table 2:** Alternative model without reaction 7: Estimated rate and dissociation constants. This table lists each reaction number alongside its corresponding biochemical equation, kinetic factors, and parameter units. Median values and literature sources are provided, with the last column showing the 95% credible intervals (CI) for the estimated kinetic factors. Note that reaction numbers five and seven were nullified in this work. Additionally, a star (*) in the 95% CI column indicates that the corresponding factor was fixed to the specified literature value. ^†^The dissociation constant *K*_*D*,4_ was recomputed separately from data presented in [9]; see supplemental S1 Text.

Reaction Number	Biochemical Equation	Kinetic Factor	Units	Median/Literature	95% CI
1	$E+S\overset{k_{1}}{\underset{k_{-1}}{⇌}}E:S$	*k*_+1_	(nM)^−1^s^−1^	0.51	(9.43 × 10^−2^, 0.96)
		*k*_−1_	s^−1^	103.93	(5.28, 211.18)
		*K*_*M*_	nM	238 [9]	*
		*K*_*D*,1_	nM	204.02	(56.16, 220.85)
2	$E:S\overset{k_{2}}{\to }E:P$	*k*_+2_	s^−1^	17.33	(13.75, 20.31)
3	$E:P\overset{k_{-3}}{\underset{k_{3}}{⇌}}E+P$	*k*_+3_	(nM)^−1^s^−1^	7.42 × 10^−2^	(3.66 × 10^−2^, 9.48 × 10^−2^)
		*k*_−3_	s^−1^	38.59	(19.02, 49.29)
		*K*_*D*,3_	nM	520 [29]	*
4	$P+I\overset{k_{4}}{\underset{k_{-4}}{⇌}}P:I$	*k*_+4_	(nM)^−1^s^−1^	3.40 × 10^−3^	(2.52 × 10^−3^, 4.54 × 10^−3^)
		*k*_−4_	s^−1^	8.94 × 10^−5^	(6.62 × 10^−5^, 1.19 × 10^−4^)
		*K*_*D*,4_	nM	2.63 × 10^−2†^	-
5	$E+P:I\overset{k_{5}}{\underset{k_{-5}}{⇌}}P:I:E$	*k*_+5_	(nM)^−1^s^−1^	-	-
		*k*_−5_	s^−1^	-	-
		*K*_*D*,5_	nM	-	-
6	$E:P+I\overset{k_{6}}{\underset{k_{-6}}{⇌}}E:P:I$	*k*_+6_	(nM)^−1^s^−1^	0.24	(0.12, 0.34)
		*k*_−6_	s^−1^	2.21 × 10^−4^	(8.37 × 10^−6^, 4.79 × 10^−4^)
		*K*_*D*,6_	nM	9.28 × 10^−4^	(3.48 × 10^−5^, 2.78 × 10^−3^)
7	$E:P:I\overset{k_{7}}{\underset{k_{-7}}{⇌}}P:I:E$	*k*_+7_	s^−1^	-	-
		*k*_−7_	s^−1^	-	-
		*K*_*R*,7_	-	-	-
8	$E+P:I\overset{k_{8}}{\underset{k_{-8}}{⇌}}E:P:I$	*k*_+8_	(nM)^−1^s^−1^	0.94	(0.76, 1.00)
		*k*_−8_	s^−1^	8.19 × 10^−4^	(2.96 × 10^−4^, 1.44 × 10^−3^)
		*K*_*D*,8_	nM	8.87 × 10^−4^	(3.24 × 10^−4^, 1.59 × 10^−3^)

Our findings show that both the original and modified schemes align well with the experimental data (see S3 Fig) and lead to equivalent results, highlighting the necessity of the DB pathway under flow (see S5, S6 and S7 Figs). However, as discussed in the Introduction, it is evident that a conformational change occurs through the Kunitz 1 domain [7, 8]. We favor the biochemical reaction based on the formation of a tight complex. This suggests that the transitions from the quaternary complex TF:VIIa:Xa:TFPI (E:P:I) to either E:P and I, or E and P:I, are inherently slow. Thus, neither reaction 4 nor reaction 6 alone can account for the slow and tight binding required to explain the observed data. As discussed in the model development, this assumption also aligns with experimental studies on the inhibition of TFPI by Xa (see [27]), suggesting that while a weak complex initially forms, there is a subsequent transition to a more stable complex.

#### Strength of product inhibition

Previous research has indicated stronger product inhibition than observed with our current model [11]. The study posited that product Xa exhibits the same binding affinity as substrate X when interacting with the enzyme. In contrast, our exploration of this model, particularly under varying flow conditions, revealed that product inhibition was not evident even when the dissociation constants *K*_*D*,1_ and *K*_*D*,3_ were made equivalent. Next we determine how much *K*_*D*,3_ must vary for product inhibition to achieve similar effects to those of direct binding.

**Fig 6:**
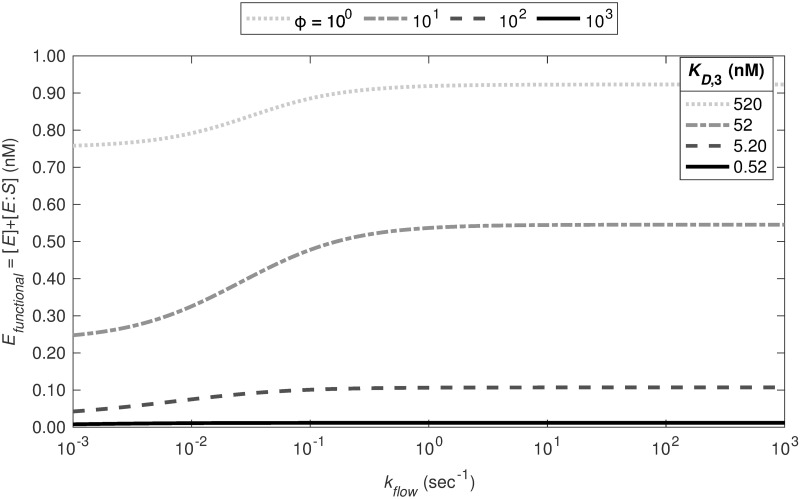
Inhibition of factor X activation is weak in the absence of TFPI. To study product inhibition we fix the forward reaction rate to the median value, *k*_+3_ = 0.16 (nM)^−1^s^−1^ and scale the median value of the reverse reaction by *ϕ*, *k*_−3_ = 81.2 ⋅ *ϕ*^−1^ s^−1^. This scales the dissociation constant *K*_*D*,3_ to 520, 52, 5.2, and 0.52 nM, respectively.

In Fig 6, we analyze the steady-state inhibition of the functional enzyme via the No TFPI pathway, focusing on the necessary level of product/enzyme dissociation for product inhibition to match the efficacy of the direct binding (DB) pathway. The dotted line represents the scenario previously depicted in Fig 5, employing median reaction rates for the third reaction as listed in Table 1: *k*_3_ = 0.16 (nM)^−1^s^−1^ and *k*_−3_ = 81.2 s^−1^. This setup corresponds to a dissociation constant (*K*_*D*_) of 520 nM. By progressively scaling down *k*_−3_ by factors of ten, we found that, with our model, the dissociation constant would need to diminish by ∼ 10^3^ to achieve an inhibition effect on par with that of the DB pathway. This scaling of the off-rate would result in a much smaller *K*_*D*_ than those reported in the literature (520 to 1773 nM, as given in [17–21, 29] and S1 Table). We repeated this analysis with the median rates for the alternate model and obtained similar results (see S8 Fig).

## Discussion and conclusion

In this study, we re-evaluated a set of experiments and proposed a kinetic scheme for the inhibition of factor X activation by TFPI. Our findings strongly support the direct binding of TFPI to the TF:VIIa:Xa complex and demonstrate that strong TFPI inhibition under flow is possible when this direct mechanism is included. We discuss our findings in the context of other studies on TFPI inhibition and its broader implications for coagulation. We also close with suggestions for advancing coagulation research through open science.

As noted in the introduction, we are not the first researchers to consider the proposed scheme with this data set. Pantaleev et al. [10] examined the same experimental results and model from Baugh [9] but reached different conclusions. They found that the model without direct binding was insufficient to match Baugh’s data and proposed a model with two additional mechanisms: direct binding of TFPI and displacement of the X substrate from TF:VIIa by the Xa:TFPI complex (see Scheme 3 in [10]). However, there is no external support for substrate displacement, leading us to conclude that the simpler formulation with direct binding is more likely.

Several differences between our work and the prior study explain our different conclusions on the sufficiency of the Baugh scheme. First, we used an adaptive Metropolis approach to parameter inference, allowing us to explore the parameter space globally. Second, we considered the likelihood of the data from both experiments simultaneously, whereas Pantaleev used a serial approach, fitting parameters from one experiment before proceeding to the next. They constrained their parameter space by assuming that the association and dissociation between X and TF:VIIa are the same as Xa and TF:VIIa. For the unknown hypothetical parameters, they considered only a narrow range of values (*k*_+7_ ∈ (0, 16.67 × 10^−3^) nM^−1^s^−1^ and *k*_+8_ ∈ (0, 3.33 × 10^−3^) nM^−1^s^−1^). Lastly, in their final comparison between data and model (Figure 2 in [10]), they did not allow reverse reactions for reaction 6 and did not include reactions 7 and 8 (forward and reverse rates for both were set to 0). They reported an inability to obtain the correct ordering of curves for the pre-incubation experiment, but increasing *k*_8_ above 0.10 nM^−1^s^−1^ with all other parameters the same would have produced the correct ordering of curves. Thus, they did not explore the full range of feasible behavior of this scheme over reasonable parameter values.

After demonstrating that the scheme with direct and indirect binding posed by Baugh plausibly fits the data, we studied the mechanisms more closely. Our inference required both direct and indirect mechanisms to simultaneously fit both static experiments. Additionally, as shown in our study with flow (see Fig 5), the inclusion of the direct binding mechanism results in strong inhibition over six orders of flow rates.

We are not the first mathematical study to closely examine direct and indirect mechanisms of TFPI inhibition of factor Xa. Fogelson and Tania [11] included both pathways but concluded that the dominant mechanism of inhibition of factor X activation was product inhibition and that flow itself was more important than TFPI for inhibition. Because [11] did not provide kinetic rates or code for their model, it is not possible to replicate their work. However, we conclude that their study did not explore the full capacity of TFPI inhibition and had other limitations. First, they assumed the same rates of binding of X to TF:VIIa and Xa to TF:VIIa. We followed [29], who reported different affinities for these reactions (*K*_*D*,1_ = 230 nM and *K*_*D*,3_ = 520 nM, respectively, for X and Xa). In their study, Fogelson and Tania varied these common *K*_*D*_ values together by a constant 0 < *α* ≤ 1, investigating the behavior of a mathematical model of the full coagulation network under flow (described in [32]). They reported the thrombin concentration at 10 minutes with a shear rate of 100 s^−1^ for different initial densities of TF with and without TFPI present, observing that the thrombin concentration was significantly impacted by *α*. Decreasing *α* increases the time that X and Xa remain in a complex with TF:VIIa, reducing the thrombin concentration (see Figure 10 in [11]). However, they found that thrombin behavior in the model was the same with or without TFPI. This does not rule out TFPI as an inhibitor, especially since we cannot verify what rates of TFPI inhibition were included in their scheme. Indeed, decreasing the *K*_*D*_ increases the capacity of X and Xa to occupy TF:VIIa, blocking the system’s ability to create additional Xa. This new model of TFPI regulation of Xa activation has the potential to influence other parts of the coagulation pathway, in particular through the complex between Xa, Va and TFPI. In order to fully explore the downstream effects that could propagate to the regulation of thrombin, this scheme should be incorporated into a full-model of static coagulation such as the ones described in [6].

Another study by Shibeko et al. [36] developed a mechanistic mathematical model of coagulation under flow, including the model of TFPI inhibition reported by [10]. These researchers concluded that the lag time of thrombin under flow was sensitive to factor VII activation and TFPI-mediated inhibition. While remarking on the source of disagreement between models on the role of TFPI, the authors wrote, “We suppose this difference was caused by different ways of modeling TFPI.” We agree with these conclusions and anticipate that our new mathematical support for the simplified direct and indirect binding scheme for TFPI inhibition brings a new appreciation for studying TFPI’s role in coagulation.

A challenge we faced was that the original data were not available, requiring us to manually extract data points from figures, introducing additional noise. While we believe our adaptive Metropolis parameter estimation approach is the best for working with such data, having access to the actual data would have been preferable. We suggest that researchers publish their experimental data in formats that facilitate quantitative comparisons and parameter estimation. Additionally, due to potential parameter identifiability and dependency issues, mathematical modelers should consider Bayesian approaches to inference, incorporating dependency and prior knowledge of rates. Furthermore, static coagulation assays occur in the presence of lipid, so modelers should control for lipid dependence by being cognizant of the specific preparations used in experimental studies or explicitly incorporating lipid surfaces into the reaction scheme.

In conclusion, our study provides new insights into the mechanisms of TFPI-mediated inhibition of factor X activation, demonstrating that both direct and indirect pathways are essential to replicate experimental observations. Moreover, the direct pathway is essential for TFPI inhibition especially under flow. Incorporating this scheme into larger models of coagulation offers the potential for a greater understanding of TFPI’s role in coagulation. Although there may be challenges in accessing published data, our work demonstrates the power of applying new mathematical approaches to existing data. Given the abundance and increasing amount of biological data available, we anticipate there is significant potential for new discoveries.

## Methods

In this section, we discuss our approach to applying the likelihood formulation for estimating parameters while incorporating bounds and relationships between rates derived from established biochemical knowledge.

### Likelihood formulation

Our formulation in Eq (5) computes the likelihood of observing the experimental measurements of factor X activation—denoted by A and presented in supplementary S1 Text—using our mathematical model and kinetic rates ***θ*** (see Table 1). The right-hand side of Eq (5) shows that the total likelihood is the product of the likelihoods of all observations from both experiments. Experiment one contains eight replicates with twelve non-zero measurements, yielding 96 measurements (*C*_1_ = 96), while experiment two contains four replicates with twelve measurements, yielding 48 measurements (*C*_2_ = 48). This likelihood formulation, together with our adaptive Metropolis approach, allows for sampling from the distribution of the kinetic rates of interest (see S1 and S2 Figs and the Estimation of Kinetic Rate Constants section).

$$\begin{array}{c} \begin{array}{cc} L(\theta |A) & =L(\theta |\text{Experiment}\;\text{1}\;\text{Data})\cdot L(\theta |\text{Experiment}\;\text{2}\;\text{Data}),\\ & =\prod_{i=1}^{C_{1}}L\left(\theta |A_{1}\left(t_{i}\right)\right)\cdot \prod_{i=1}^{C_{2}}L\left(\theta |A_{2}\left(t_{i}\right)\right),\\ & =\prod_{k=1}^{2}\prod_{i=1}^{C_{k}}L\left(\theta |A_{k}\left(t_{i}\right)\right). \end{array} \end{array}$$ (5)

As aforementioned, the likelihood of the experimental data L(θ|A) is the product of the likelihoods of all measurements and their associated model predictions given kinetic rates ***θ***. The likelihood of a single observation is computed assuming a proportional error model Eq (6), where for a single observation $A(t_{i})$ at time *t*_*i*_ and the model prediction *μ*(*t*_*i*_|***θ***), N is the normal distribution with mean *μ*(*t*_*i*_|***θ***) and a standard deviation proportional to the model prediction *σ* ⋅ *μ*(*t*_*i*_|***θ***). The *σ* ⋅ *μ*(*t*_*i*_|***θ***) term captures the observed heteroscedasticity in the data with increased magnitude of factor X measurements (see supplementary figures Figs A and B in S1 Text). The model factors in ***θ*** include the kinetic rate constants, *k*_1_, *k*_−1_, *k*_2_, *k*_+3_, *k*_−3_, …*k*_+8_, *k*_−8_, the standard deviation term *σ*, and the initial conditions depending on whether the model prediction is for experiment one or experiment two, [*E*]_0_, [*S*]_0_, …, [*P*:*I*:*E*]_0_. See the Results section for an explanation of experiments and their initial conditions.

$$L(\theta |A_{k}\left(t_{i}\right))=N(A_{k}\left(t_{i}\right);\mu \left(t_{i}|\theta \right),\sigma \cdot \mu \left(t_{i}|\theta \right)).$$ (6)

### Estimates of kinetic rate constants

To estimate rate constants, we follow the convention of quantifying forward and backward reactions, setting the lower bound of all rate constants to zero. The upper bound of diffusion-limited enzyme substrate reactions is set to 1 (nM)^−1^s^−1^ [37], and the upper limit for the reverse reactions is set to 500 s^−1^. To account for the rapid conversion of the E:P:I complex to the more stable P:I:E complex, we set the upper bound for the forward rate constant *k*_+7_ to 500 s^−1^ and the upper limit for the reverse conformational rate constant *k*_−7_ to 10^−2^ s^−1^. We found that setting a higher bound on *k*_−7_ still led to *k*_−7_ ≪ *k*_+7_ and equivalent results. We use the Michaelis-Menten relationship [38]:

$$\begin{array}{c} K_{M}=\frac{k_{-1}+k_{2}}{k_{1}} \end{array}$$ (7)

to establish bounds on the rate constants *k*_1_ and *k*_2_, assuming a known value for *K*_*M*_ (see Table 1). Using the known value for *K*_*M*_ and the estimated values of *k*_1_ and *k*_2_, we set the reverse reaction rate *k*_−1_ = *K*_*M*_ ⋅ *k*_1_ − *k*_2_. Requiring *k*_−1_ ≥ 0, we find that *k*_1_ ≥ *k*_2_/*K*_*M*_, with an upper bound of 1 (nM)^−1^s^−1^. This also implies that *k*_2_/*K*_*M*_ ≤ 1 (nM)^−1^s^−1^, thus *k*_2_ ≤ *K*_*M*_ s^−1^.

To estimate the posterior distributions of the unknown rate constants (see Fig B in S1 Text), we follow four steps: (i) a pre-exploration of the parameter space using a uniform prior on the set of ranges previously described, (ii) application of the Metropolis algorithm (MA), (iii) application of the adaptive Metropolis algorithm (AM), and (iv) post-processing of chain iterations to reduce autocorrelation among estimates. In the uniform pre-exploration of the parameter space, we compute the likelihood as in Eq (5) using 10^6^ Latin hypercube samples (LHS) and apply the bounds and relationships previously discussed. Following [39], we use our likelihood formulation presented in Eq (5) and the normal distribution as our proposal distribution to apply a two-step random walk Metropolis algorithm to estimate the target distributions. In the application of MA, which is an initial random walk exploration of the parameter space, we first use the top 500 LHS parameter sets associated with the top 500 likelihood values out of the 10^6^ values to compute an initial set of parameters ***θ***_0_ and an initial diagonal variance matrix ***V***. Assuming a normal prior, we compute 10^5^ iterations of the MA. In the application of the AM, we use the 10^5^ MA iterations to compute the covariance matrix ***C*** and use the last MA parameter set ***θ***_10^5^_ as the initial AM parameter set, computing 6 ⋅ 10^6^ iterations with a normal prior. Lastly, to reduce the autocorrelation between iterations of the combined MA and AM chain iterations to less than 5% (as discussed in [40]), we drop the first 10^5^ MA iterations and then take every 100th sample of the chain. This leaves 6 ⋅ 10^4^ estimates per chain, which we use for the analyses in this work.

## Supporting information

S1 Text Extraction of experimental data and re-estimating *K*_*D*,4_ from data in [9]. Fig A. Progress Curves of Factor X Activation Data Extracted From Figures 2A and 3B of [9]. Fig B. Inhibition of Factor Xa by TFPI. Table A. Experiment One Data Extracted From Figure 2A of [9]. Table B. Experiment Two Data Extracted From Figure 3B of [9]. Table C. Data Extracted From Figure 4A in [9].
(PDF)

S1 Fig Posterior distribution of rate constants and proportional error term. Posterior distributions are formed from samples obtained through application of the Adaptive Metropolis algorithm, as detailed in the main text, and presented within a 99% credible interval window.
(PDF)

S2 Fig Posterior distribution of dissociation constants and kinetic ratio. Posterior distributions are formed from samples obtained through application of the adaptive Metropolis algorithm, as detailed in the main text, and presented within a 99% credible interval window.
(PDF)

S3 Fig Alternative model without a stable complex: Fit to factor X activation curves. Uncertainty in model predictions: Median, 70%, 90%, and 99% credible intervals about the median. (A) Factor X (170 nM) activated by VIIa:TF (0.032 to 1.024 nM) in the presence of TFPI (2.4 nM) (see [9]). (B) Factor X (170 nM) activated by VIIa:TF (0.128 nM) in the presence of TFPI (2.4 nM) preincubated with factor Xa (0.00 to 1.00 nM) (see [9]).
(PDF)

S4 Fig Alternative model without a stable complex: Posterior distribution of rate constants and proportional error term. Posterior distributions are formed from samples obtained through application of the adaptive Metropolis algorithm, as detailed in the main text, and presented within a 99% credible interval window.
(PDF)

S5 Fig Alternative model without a stable complex: Posterior distribution of dissociation constants. Posterior distributions are formed from samples obtained through application of the adaptive Metropolis algorithm, as detailed in the main text, and presented within a 99% credible interval window.
(PDF)

S6 Fig Alternative model without a stable complex: Functional enzyme over time by flow rate and inhibition pathway. Concentration of functional enzyme over time by pathway of inhibition, for (A) low flow (*k*_*flow*_ = 10^−3^ s^−1^), (B) medium flow (*k*_*flow*_ = 10^0^ s^−1^), and (C) high flow (*k*_*flow*_ = 10^3^ s^−1^). *See* Fig 4 *in the main text for comparison*.
(PDF)

S7 Fig Alternative model without a stable complex: Functional enzyme at steady state over flow rate. For each inhibition pathway: No TFPI (NI), Direct Binding (DB), Indirect Binding (IB), and both the Direct and Indirect Binding (DIB), the steady state concentration of functional enzyme is presented for flow rates from 10^−3^ (Low Flow) to 10^3^ sec^−1^ (High Flow). *See* Fig 5 *in the main text for comparison*.
(PDF)

S8 Fig Alternative model without a stable complex: Inhibition of factor X activation is weak in the absence of TFPI. To study product inhibition we fix the forward reaction rate to the median value, *k*_+3_ = 7.42 ⋅ 10^−2^ (nM)^−1^s^−1^ and scale the median value of the reverse reaction by *ϕ*, *k*_−3_ = 38.9 ⋅ *ϕ*^−1^ s^−1^. This scales the dissociation constant *K*_*D*,3_ to 520, 52, 5.2, and 0.52 nM, respectively. *See* Fig 6 *in the main text for comparison*.
(PDF)

S1 Table Kinetic rates of interest in previous models in thrombin generation models considered in [6]. (PDF)
