# Supplementary material for: A new look at TFPI inhibition of factor X activation
Source: PLoS Comput Biol. 2024 Nov 15;20(11):e1012509. doi: 10.1371/journal.pcbi.1012509 (PMC11567595; doi:10.1371/journal.pcbi.1012509)
Supplement: S1 Text — Fig A. Progress Curves of Factor X Activation Data Extracted From Figures 2A and 3B of [9]. Fig B. Inhibition of Factor Xa by TFPI. Table A. Experiment One Data Extracted From Figure 2A of [9]. Table B. Experiment Two Data Extracted From Figure 3B of [9]. Table C. Data Extracted From Figure 4A in [9]. (PDF) [file pcbi.1012509.s001.pdf]

S1 Text

Extraction of Experimental Data From [1]

The data used in this work was extracted from **Fig 2A** and **3B** of [1] by uploading the figures from the original paper to the online tool for data extraction [2]. Using this tool, the x and y axes were first established, and then a set of factor X activation points was formed for each initial condition by selecting individual points. This online tool then returned the data presented in **Fig A**(A) as eight separate datasets and those in **Fig A**(B) as four datasets (see **Table A** and **Table B**).

These datasets, along with our model predictions, were used to compute the likelihood of various kinetic rates in the application of our Metropolis approach, aiming to provide a comprehensive explanation of the observed data. The Methods section of the main text explains the process of how we utilized the data from both experiments, detailing the steps and considerations involved. Additionally, the detailed procedures and outcomes of each experiment are thoroughly presented in the Results section to provide a clear understanding of our findings.

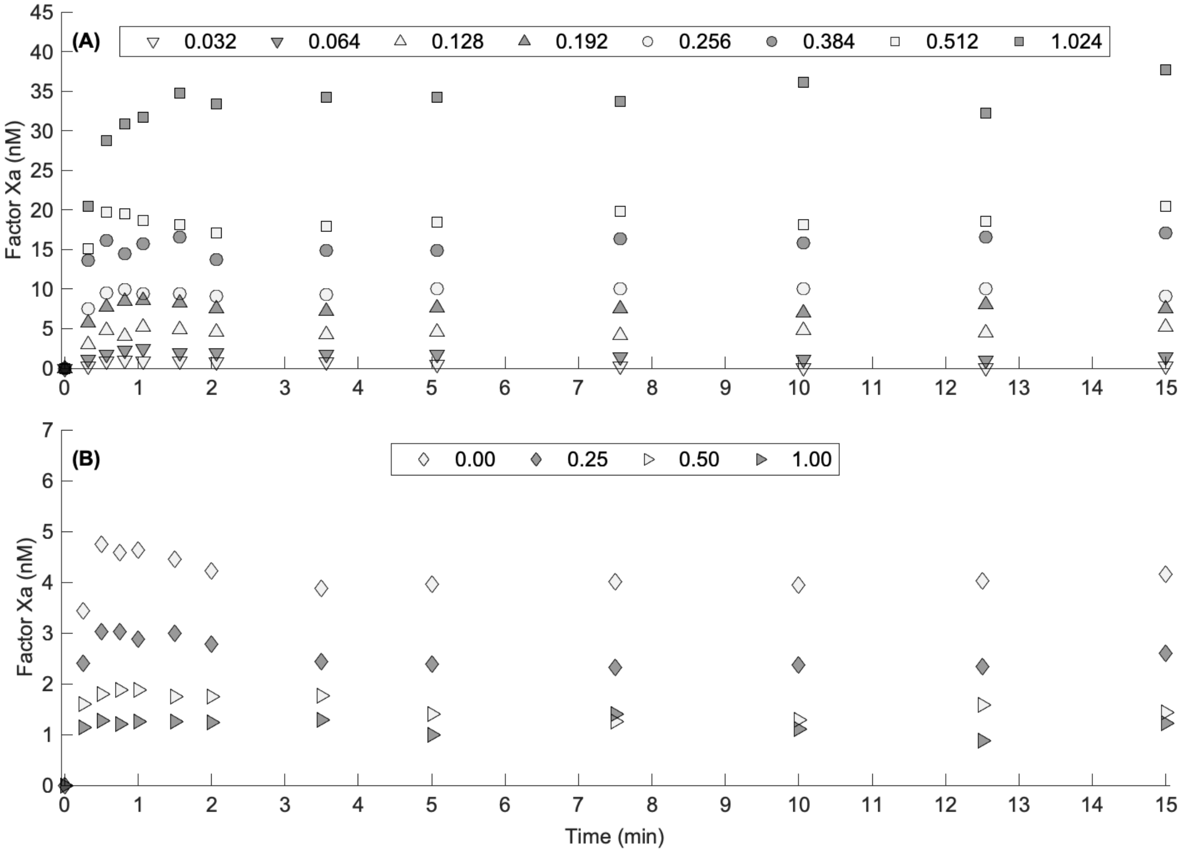

Fig A. Progress Curves of Factor X Activation Data Extracted From Figures 2A and 3B of [1]. (A) Progress curves of factor X (170 nM) activated by VIIa−TF (0.032 to 1.024 nM) in the presence of TFPI (2.4 nM) from Figure 2A of [1]. (B) Progress curves of factor X (170 nM) activated by VIIa−TF (0.128 nM) in the presence of TFPI (2.4 nM) preincubated with factor Xa (0.00 to 1.00 nM) from Figure 3B of [1].

$\boldsymbol{E_{tot}(0)}$ **(nM)**	**Time (sec)**												
	0	19	34	49	64	94	124	214	304	454	604	753	900
	**Factor Xa (nM)**												
0.032	0.00	0.30	0.87	0.96	0.91	0.87	0.74	0.74	0.52	0.30	0.04	0.09	0.26
0.064	0.00	1.09	1.78	2.26	2.52	1.96	1.96	1.74	1.70	1.43	1.09	1.04	1.43
0.128	0.00	3.04	4.83	4.09	5.22	4.87	4.61	4.30	4.61	4.17	4.78	4.48	5.17
0.192	0.00	5.70	7.70	8.43	8.52	8.22	7.57	7.22	7.61	7.52	7.04	8.09	7.57
0.256	0.00	7.48	9.48	9.91	9.43	9.43	9.13	9.26	10.00	10.09	10.04	10.04	9.13
0.384	0.00	13.65	16.13	14.43	15.74	16.61	13.70	14.83	14.87	16.35	15.83	16.52	17.13
0.512	0.00	15.04	19.70	19.52	18.70	18.09	17.09	17.91	18.48	19.83	18.09	18.61	20.43
1.024	0.00	20.43	28.78	30.83	31.65	34.74	33.35	34.17	34.26	33.70	36.09	32.22	37.65

Table A. Experiment One Data Extracted From Figure 2A of [1]. Progress curves of factor X (170 nM) activated by VIIa−TF (1.024 to 0.032 nM) in the presence of TFPI (2.4 nM).

$\mathbf{Xa}_{pre}\mathbf{(0)}$ **(nM)**	**Time (sec)**												
	0	15	30	45	60	90	120	210	300	450	600	750	900
	**Factor Xa (nM)**												
0.000	0.00	3.44	4.74	4.59	4.63	4.45	4.22	3.88	3.96	4.01	3.94	4.03	4.15
0.250	0.00	2.40	3.02	3.03	2.87	2.99	2.78	2.44	2.38	2.32	2.37	2.34	2.60
0.500	0.00	1.60	1.80	1.87	1.87	1.75	1.75	1.76	1.40	1.26	1.28	1.59	1.44
1.000	0.00	1.14	1.27	1.20	1.26	1.25	1.23	1.29	1.00	1.40	1.11	0.87	1.22

Table B. Experiment Two Data Extracted From Figure 3B of [1]. Progress curves of factor X (170 nM) activated by VIIa−TF (0.128 nM) in the presence of TFPI (2.4 nM) preincubated with factor Xa (0.00 to 1.00 nM).

Re-estimating $K_{D,4}$ From Data in [1]

We re-computed $K_{D,4}$ using data extracted from Figure 4A in [1] (see **Table C**), as described in the previous section. Using the kinetic rates $k_4 = 9.0 \times 10^{-4}$ $(\text{nM})^{-1}\text{s}^{-1}$ and $k_{-4} = 3.6 \times 10^{-4}$ s^{-1} (column 1 of Table 1 in [1]), the dissociation constant is $K^*_{D,4} = 0.40$ nM. However, given equation four of Table 1 in the main text, and reproduced here as Eq. (1), we can use the law of mass action to write down the associated system of ordinary differential equations (Eq. (2)). To find the equilibrium points, we set Eq. (2) equal to zero and solve the system for [Xa], leading to a quadratic equation in [Xa] and resulting in Eq. (3) where $K_{D,4} = \frac{k_{-4}}{k_4}$.

Using Eq. (3), we select the solution that results in positive concentrations of Xa ($[\text{Xa}]_+$). Applying a proportional error model of the form $Y_{ij} = D_{ij} + \epsilon D_{ij}$, where D_{ij} is the factor Xa given the ith initial concentration of TFPI and the jth initial concentration of Xa, and Y_{ij} is the value given by Eq. (3), we compute the associated residual sum of squares (RSS). Using MATLAB's `fmincon`, we determine an optimal dissociation constant of $K_{D,4} = 0.0263$ nM that minimizes the RSS.

With the re-computed $K_{D,4} = 0.0263$ nM and $k_4 = 9.0 \times 10^{-4}$ $(\text{nM})^{-1}\text{s}^{-1}$, we re-compute the reverse reaction kinetic rate constant, $k_{-4} = 2.367 \times 10^{-5}$ nM. In **Fig B**, we show the difference between fits using the dissociation constant $K^*_{D,4} = 0.40$ nM [1] and our re-computed $K_{D,4} = 0.0263$ nM. From **Fig B**, we observe that our re-computed $K_{D,4}$ best explains the residual factor Xa as TFPI increases (black curves).

$$[\mathrm{Xa}] + [\mathrm{TFPI}] \underset{k_{-4}}{\overset{k_4}{\rightleftharpoons}} [\mathrm{Xa{:}TFPI}] \tag{1}$$

$$\begin{aligned} \frac{d[\mathrm{Xa}]}{dt} &= -k_4[\mathrm{Xa}][\mathrm{TFPI}] + k_{-4}[\mathrm{Xa{:}TFPI}] \\ \frac{d[\mathrm{TFPI}]}{dt} &= -k_4[\mathrm{Xa}][\mathrm{TFPI}] + k_{-4}[\mathrm{Xa{:}TFPI}] \\ \frac{d[\mathrm{Xa{:}TFPI}]}{dt} &= +k_4[\mathrm{Xa}][\mathrm{TFPI}] - k_{-4}[\mathrm{Xa{:}TFPI}] \end{aligned} \tag{2}$$

$$[\mathrm{Xa}]_{+,-} = \frac{-([\mathrm{TFPI}]_0 - [\mathrm{Xa}]_0 + K_{D,4}) \pm \sqrt{([\mathrm{TFPI}]_0 - [\mathrm{Xa}]_0 + K_{D,4})^2 + 4[\mathrm{Xa}]_0 K_{D,4}}}{2}. \tag{3}$$

**$[\mathrm{TFPI}]_0$ (nM)**	**Rate ($\Delta_{405} \times 10^{-3}/min$)**	
	**$[\mathrm{Xa}]_0 = 0.10$ nM**	**$[\mathrm{Xa}]_0 = 0.20$ nM**
0.000	4.033	7.771
0.021	3.491	7.083
0.044	2.927	6.375
0.072	2.233	5.312
0.094	1.930	4.812
0.118	1.583	4.229
0.138	1.366	3.688
0.161	1.062	3.292
0.183	0.954	2.854
0.209	0.759	2.417
0.232	0.759	2.125
0.255	0.672	1.750
0.275	0.542	1.562
0.316	0.607	1.188
0.348	0.369	1.062
0.387	0.304	0.812
0.426	0.260	0.750
0.460	0.304	0.729
0.497	0.217	0.583
0.536	0.217	0.562
0.568	0.195	0.479
0.608	0.152	0.417
0.646	0.173	0.396
0.680	0.173	0.375

Table C. Data Extracted From Figure 4A in [1]. Inhibition of factor Xa activity by initial concentration of TFPI. The experiment consists of mixing initial concentrations of $[\mathrm{Xa}]_0 = 0.10$ nM and $[\mathrm{Xa}]_0 = 0.20$ nM with increasing concentrations of TFPI (0.00 to 680 nM) and measuring the residual factor Xa concentration after prolonged incubation in both cases. See [1] for more detail.

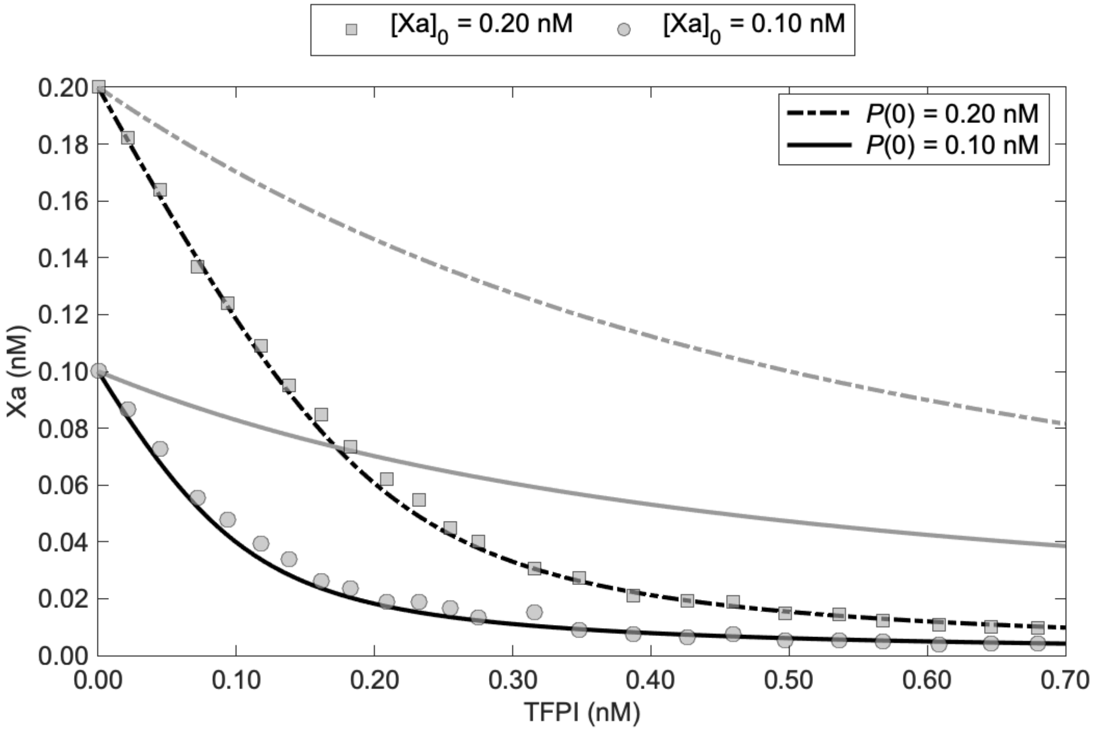

Fig B. Inhibition of Factor Xa by TFPI. The residual factor Xa as TFPI increases for initial concentrations of Xa = 0.20 nM (circles) and nM Xa = 0.10 (squares) following prolonged incubation. The *black curves* are computed using Eq. (2) and the re-computed rates k_4 and k_{-4} using $K_{D,4} = 0.0263$ nM, and the *gray curves* are computed using the rates presented in [1].

References

[1] Robert J. Baugh, George J. Broze, and Sriram Krishnaswamy. Regulation of extrinsic pathway factor Xa formation by tissue factor pathway inhibitor. *Journal of Biological Chemistry*, 273(8):4378–4386, 1998.

[2] Kristian Pontoppidan Larsen. Graphreader, 2024. Accessed: 2024-04-09.
