# Supplementary material for: A new look at TFPI inhibition of factor X activation
Source: PLoS Comput Biol. 2024 Nov 15;20(11):e1012509. doi: 10.1371/journal.pcbi.1012509 (PMC11567595; doi:10.1371/journal.pcbi.1012509)
Supplement: S1 Fig — Posterior distributions are formed from samples obtained through application of the Adaptive Metropolis algorithm, as detailed in the main text, and presented within a 99% credible interval window. (PDF) [file pcbi.1012509.s002.pdf]

S1 Fig

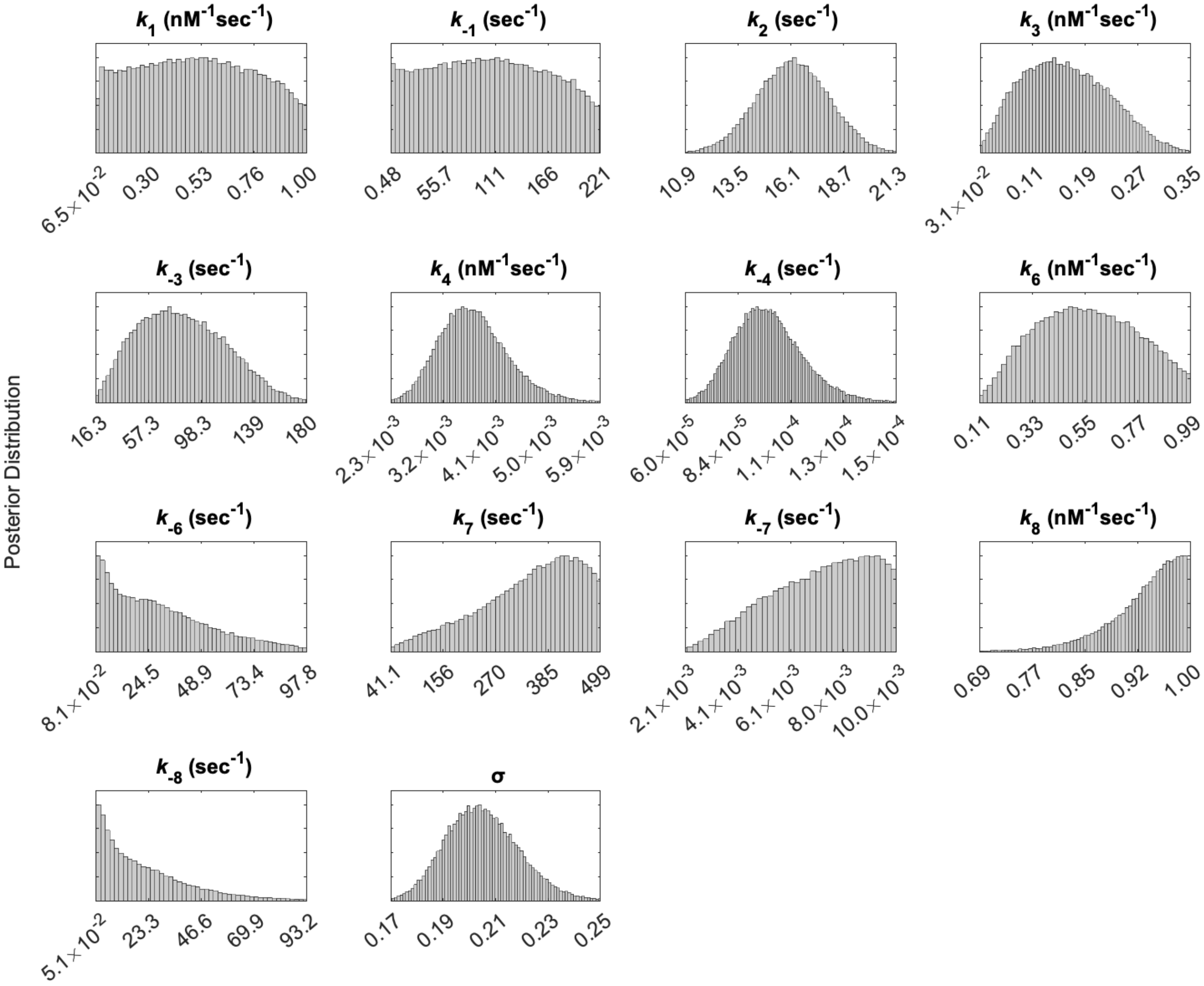

Posterior Distribution of Rate Constants and Proportional Error Term. Posterior distributions are formed from samples obtained through application of the Adaptive Metropolis algorithm, as detailed in the main text, and presented within a 99% credible interval window.
