# Supplementary material for: A new look at TFPI inhibition of factor X activation
Source: PLoS Comput Biol. 2024 Nov 15;20(11):e1012509. doi: 10.1371/journal.pcbi.1012509 (PMC11567595; doi:10.1371/journal.pcbi.1012509)
Supplement: S3 Fig — Uncertainty in model predictions: Median, 70%, 90%, and 99% credible intervals about the median. (A) Factor X (170 nM) activated by VIIa:TF (0.032 to 1.024 nM) in the presence of TFPI (2.4 nM) (see [9]). (B) Factor X (170 nM) activated by VIIa:TF (0.128 nM) in the presence of TFPI (2.4 nM) preincubated with factor Xa (0.00 to 1.00 nM) (see [9]). (PDF) [file pcbi.1012509.s004.pdf]

S3 Fig

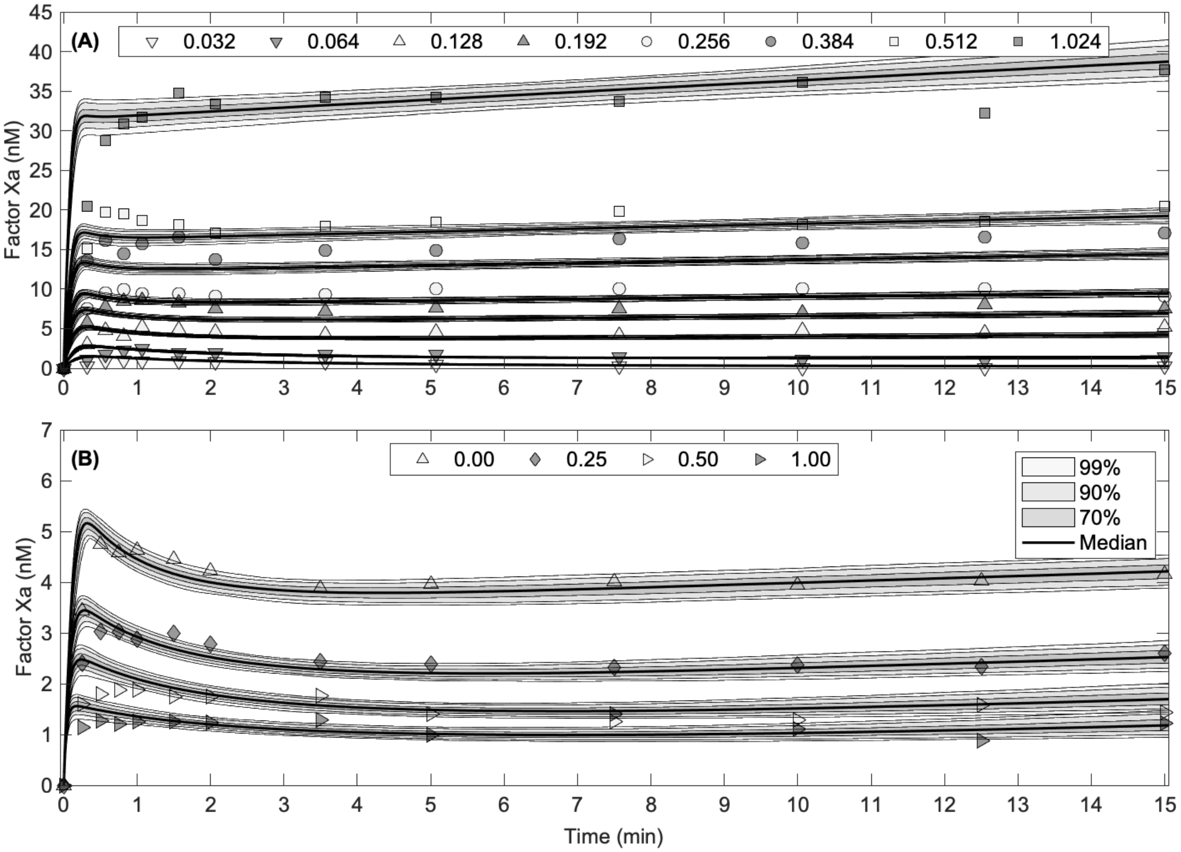

Alternative Model Without a Stable Complex: Fit to Factor X Activation Curves. Uncertainty in model predictions: Median, 70%, 90%, and 99% credible intervals about the median. (A) Factor X (170 nM) activated by VIIa:TF (0.032 to 1.024 nM) in the presence of TFPI (2.4 nM) (see [1]). (B) Factor X (170 nM) activated by VIIa:TF (0.128 nM) in the presence of TFPI (2.4 nM) preincubated with factor Xa (0.00 to 1.00 nM) (see [1]).

References

[1] Robert J. Baugh, George J. Broze, and Sriram Krishnaswamy. Regulation of extrinsic pathway factor Xa formation by tissue factor pathway inhibitor. *Journal of Biological Chemistry*, 273(8):4378–4386, 1998.
