# Supplementary material for: A new look at TFPI inhibition of factor X activation
Source: PLoS Comput Biol. 2024 Nov 15;20(11):e1012509. doi: 10.1371/journal.pcbi.1012509 (PMC11567595; doi:10.1371/journal.pcbi.1012509)
Supplement: S5 Fig — Posterior distributions are formed from samples obtained through application of the adaptive Metropolis algorithm, as detailed in the main text, and presented within a 99% credible interval window. (PDF) [file pcbi.1012509.s006.pdf]

# S5 Fig

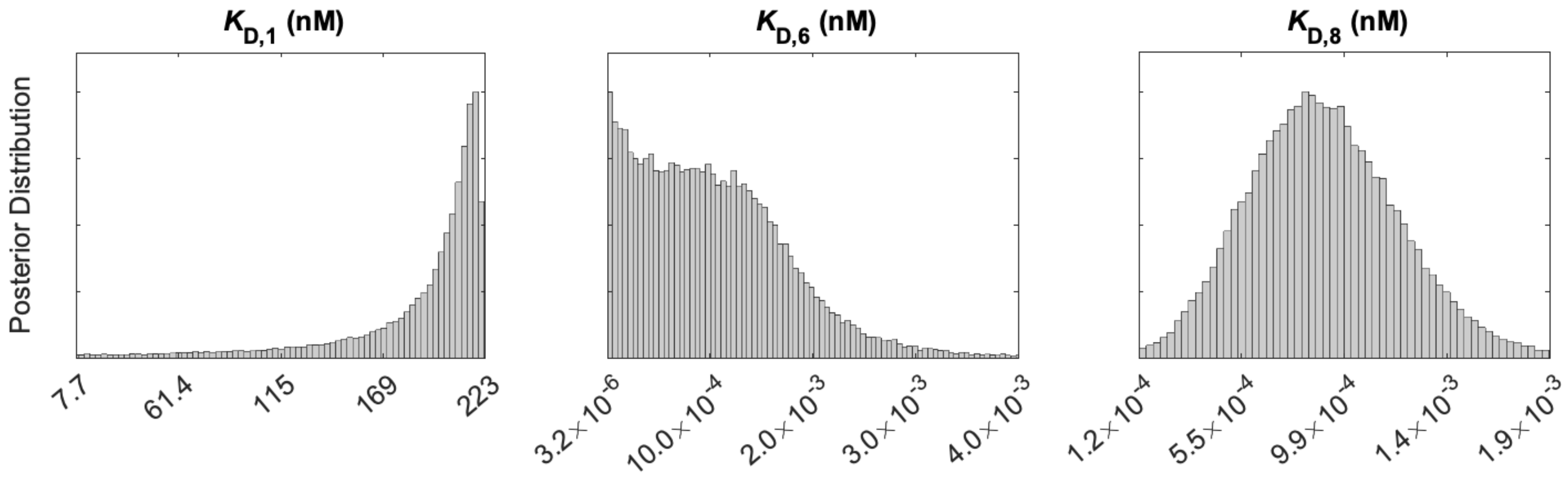


**Alternative Model Without a Stable Complex: Posterior Distribution of Dissociation Constants**. Posterior distributions are formed from samples obtained through application of the adaptive Metropolis algorithm, as detailed in the main text, and presented within a 99% credible interval window.
