# Supplementary material for: A new look at TFPI inhibition of factor X activation
Source: PLoS Comput Biol. 2024 Nov 15;20(11):e1012509. doi: 10.1371/journal.pcbi.1012509 (PMC11567595; doi:10.1371/journal.pcbi.1012509)
Supplement: S6 Fig — Concentration of functional enzyme over time by pathway of inhibition, for (A) low flow (kflow = 10−3 s−1), (B) medium flow (kflow = 100 s−1), and (C) high flow (kflow = 103 s−1). See Fig 4 in the main text for comparison. (PDF) [file pcbi.1012509.s007.pdf]

S6 Fig

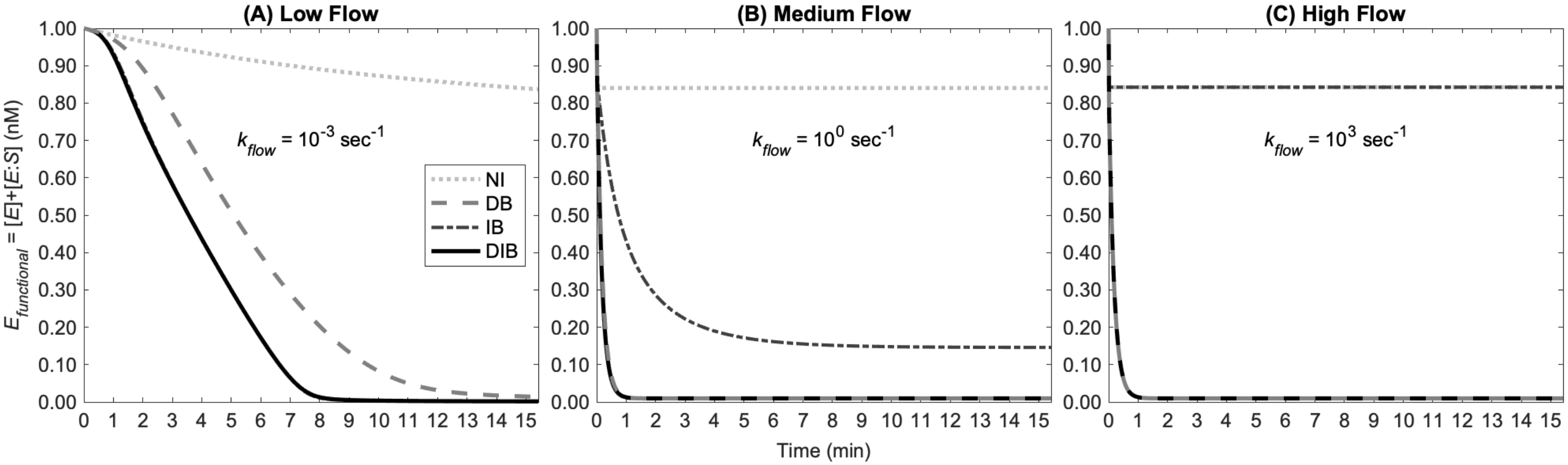

Alternative Model Without a Stable Complex: Functional Enzyme Over Time by Flow Rate and Inhibition Pathway. Concentration of functional enzyme over time by pathway of inhibition, for (A) low flow ($k_{flow} = 10^{-3}$ s^{-1}), (B) medium flow ($k_{flow} = 10^{0}$ s^{-1}), and (C) high flow ($k_{flow} = 10^{3}$ s^{-1}). *See Fig 4 in the main text for comparison.*
