# Supplementary material for: A new look at TFPI inhibition of factor X activation
Source: PLoS Comput Biol. 2024 Nov 15;20(11):e1012509. doi: 10.1371/journal.pcbi.1012509 (PMC11567595; doi:10.1371/journal.pcbi.1012509)
Supplement: S7 Fig — For each inhibition pathway: No TFPI (NI), Direct Binding (DB), Indirect Binding (IB), and both the Direct and Indirect Binding (DIB), the steady state concentration of functional enzyme is presented for flow rates from 10−3 (Low Flow) to 103 sec−1 (High Flow). See Fig 5 in the main text for comparison. (PDF) [file pcbi.1012509.s008.pdf]

S7 Fig

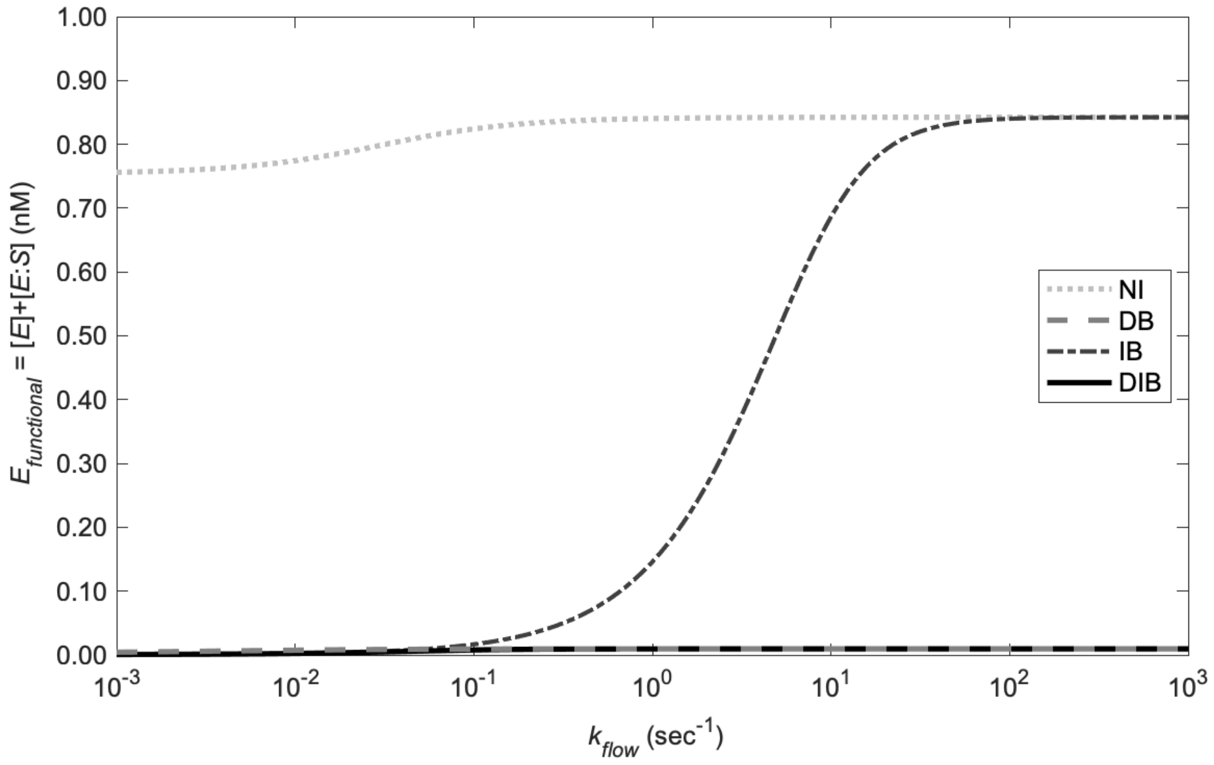

Alternative Model Without a Stable Complex: Functional Enzyme at Steady State Over Flow Rate. For each inhibition pathway: No TFPI (NI), Direct Binding (DB), Indirect Binding (IB), and both the Direct and Indirect Binding (DIB), the steady state concentration of functional enzyme is presented for flow rates from 10^{-3} (Low Flow) to 10^{3} sec^{-1} (High Flow). *See Fig 5 in the main text for comparison.*
