# Supplementary material for: A new look at TFPI inhibition of factor X activation
Source: PLoS Comput Biol. 2024 Nov 15;20(11):e1012509. doi: 10.1371/journal.pcbi.1012509 (PMC11567595; doi:10.1371/journal.pcbi.1012509)
Supplement: S8 Fig — To study product inhibition we fix the forward reaction rate to the median value, k+3 = 7.42 ⋅ 10−2 (nM)−1s−1 and scale the median value of the reverse reaction by ϕ, k−3 = 38.9 ⋅ ϕ−1 s−1. This scales the dissociation constant KD,3 to 520, 52, 5.2, and 0.52 nM, respectively. See Fig 6 in the main text for comparison. (PDF) [file pcbi.1012509.s009.pdf]

S8 Fig

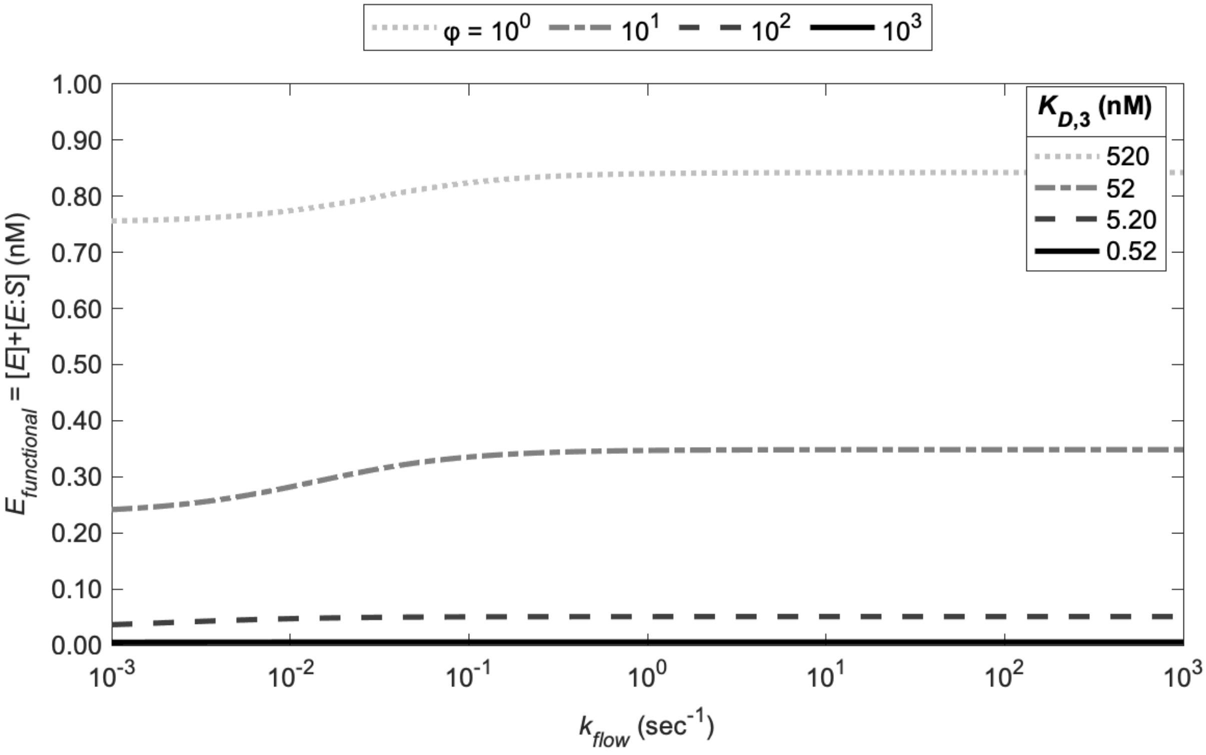

Alternative Model Without a Stable Complex: Inhibition of Factor X Activation is Weak in the Absence of TFPI. To study product inhibition we fix the forward reaction rate to the median value, $k_{+3} = 7.42 \cdot 10^{-2}$ $(\text{nM})^{-1}\text{s}^{-1}$ and scale the median value of the reverse reaction by ϕ, $k_{-3} = 38.9 \cdot \phi^{-1}$ s^{-1}. This scales the dissociation constant $K_{D,3}$ to 520, 52, 5.2, and 0.52 nM, respectively. *See Fig 6 in the main text for comparison.*
