# Supplementary material for: A new look at TFPI inhibition of factor X activation
Source: PLoS Comput Biol. 2024 Nov 15;20(11):e1012509. doi: 10.1371/journal.pcbi.1012509 (PMC11567595; doi:10.1371/journal.pcbi.1012509)
Supplement: S1 Table — (PDF) [file pcbi.1012509.s010.pdf]

S1 Tab

**Kinetic Factor**	**Units**	**[1, 2, 3]**	**[4, 5]**	**[6]**	**[7]**
k_{+3}	$(\text{nM})^{-1}\text{s}^{-1}$	2.2×10^{-2}	2.2×10^{-2}	-	-
k_{-3}	s^{-1}	19	39	1.0	1.0
$K_{D,3}$	nM	863.6	1772.7	-	-
k_{+6}	$(\text{nM})^{-1}\text{s}^{-1}$	3.2×10^{-1}	1.0×10^{-2}	-	1.0×10^{-1}
k_{-6}	s^{-1}	1.1×10^{-2}	1.0×10^{-5}	-	-
$K_{D,6}$	nM	3.4×10^{-2}	1.0×10^{-3}	-	-
$T_{1/2}$	s	6.3×10^{1}	6.9×10^{4}	-	-
k_{+4}	$(\text{nM})^{-1}\text{s}^{-1}$	9.0×10^{-4}	2.2×10^{-2}	1.0×10^{-2}	8.67×10^{-4}
k_{-4}	s^{-1}	3.6×10^{-4}	3.7×10^{-5}	1.1×10^{-3}	3.33×10^{-5}
$K_{D,4}$	nM	4.0×10^{-1}	3.77×10^{-2}	1.1×10^{-1}	3.84×10^{-1}

Kinetic Rates of Interest in Previous Models in Thrombin Generation Models Considered in [8]. This table summarizes the kinetic rates of interest used in previous models of thrombin generation using reaction numbers in the main text. [1, 2, 3] are the Chatterjee model model, Danforth model, and Hockin model; [4, 5] are the Lakshmanan model and the Brummel-Ziedins model; [6] is the Bungay model and [7] is the Panteleev model. The hyphen (-), indicates that the reaction(s) and associated rates were not considered in those models.

References

[1] Manash S Chatterjee, William S Denney, Huiyan Jing, and Scott L Diamond. Systems biology of coagulation initiation: kinetics of thrombin generation in resting and activated human blood. *PLoS computational biology*, 6(9):e1000950, 2010.

[2] Christopher M. Danforth, Thomas Orfeo, Kenneth G. Mann, Kathleen E. Brummel-Ziedins, and Stephen J. Everse. The impact of uncertainty in a blood coagulation model. *Mathematical Medicine and Biology*, 26:323–336, 5 2009.

[3] Matthew F Hockin, Kenneth C Jones, Stephen J Everse, and Kenneth G Mann. A model for the stoichiometric regulation of blood coagulation. *Journal of Biological Chemistry*, 277(21):18322–18333, 2002.

[4] Hari Hara Sudhan Lakshmanan, Aldrich Estonilo, Stéphanie E Reitsma, Alexander R Melrose, Jayaram Subramanian, Tony J Zheng, Jeevan Maddala, Erik I Tucker, David Gailani, Owen JT McCarty, et al. Revised model of the tissue factor pathway of thrombin generation: role of the feedback activation of fxi. *Journal of Thrombosis and Haemostasis*, 20(6):1350–1363, 2022.

[5] Kathleen E. Brummel-Ziedins, Thomas Orfeo, Peter W. Callas, Matthew Gissel, Kenneth G. Mann, and Edwin G. Bovill. The prothrombotic phenotypes in familial protein c deficiency are differentiated by computational modeling of thrombin generation. *PLOS ONE*, 7(9):1–10, 09 2012.

[6] Sharene D Bungay, Patricia A Gentry, and Rodney D Gentry. A mathematical model of lipid-mediated thrombin generation. *Mathematical Medicine and Biology*, 20(1):105–129, 2003.

[7] Mikhail A Panteleev, Anna N Balandina, Elena N Lipets, Mikhail V Ovanesov, and Fazoil I Ataullakhanov. Task-oriented modular decomposition of biological networks: trigger mechanism in blood coagulation. *Biophysical journal*, 98(9):1751–1761, 2010.

[8] Matt J Owen, Joy R Wright, Edward GD Tuddenham, John R King, Alison H Goodall, and Joanne L Dunster. Mathematical models of coagulation-are we there yet? *Journal of Thrombosis and Haemostasis*, 2024.
